# Detector-Stage Failures Account for Most of the Increase in the External Deficit of a Box-Prompted Glioma Segmentation Pipeline on BraTS-Africa: A Three-Arm Decomposition Across Two External Cohorts

**DOI:** 10.3390/diagnostics16203391

**Published:** 2026-10-18

**Authors:** Aykut Kaşkaya, Baha Şen

**Affiliations:** 1 Department of Computer Engineering, Ankara Yıldırım Beyazıt University, Ankara 06010, Türkiye; 2 Department of Computer Engineering, Turkish Military Academy, National Defence University, Ankara 06420, Türkiye; baha.sen@msu.edu.tr

**Keywords:** glioma, magnetic resonance imaging, brain tumor segmentation, external validation, distribution shift, promptable segmentation, foundation models, bounding-box detection, evaluation methodology, BraTS

## Abstract

**Background/Objectives:** When a box-prompted segmentation pipeline loses accuracy on new data, the detector proposing the box or a later stage may be responsible; this study measured which. **Methods:** A real-time detection transformer (RT-DETR) feeding the Segment Anything Model 2.1 was compared with a detector-free model (MedSAM3 with a low-rank adapter) on whole-tumor Dice in 158 patients with glioma: held-out Brain Tumor Segmentation (BraTS) 2020 cases (*n* = 24), the RHUH-GBM glioblastoma cohort (*n* = 39), and BraTS-Africa (*n* = 95, glioma presumed). Giving the segmenter the ground-truth box splits the Dice gap between the systems into a detector-stage term and a residual. **Results:** The detector-stage term was +0.006 Dice on held-out cases, +0.018 on RHUH-GBM, and +0.091 on BraTS-Africa. Its increase over the held-out value was +0.0854 [95% CI +0.0450, +0.1303] on BraTS-Africa (Holm-adjusted *p* < 0.001) and, after correction of a preprocessing fault, +0.0120 [−0.0062, +0.0295] on RHUH-GBM, which was not resolved. On BraTS-Africa, it came mainly from slices without a detector box. No residual change was resolved. **Conclusions:** On BraTS-Africa, the detector stage accounted for most of the increase in the pipeline’s gap; on RHUH-GBM, no increase was resolved. The decomposition should be measured per cohort.

## 1. Introduction

Glioma segmentation from multiparametric magnetic resonance imaging (MRI) is usually posed as a dense prediction. The Brain Tumor Segmentation (BraTS) benchmark [1,2,3] supplies the in-domain data used here, with its nested regions: the enhancing tumor (ET) lies inside the tumor core (TC), which lies inside the whole tumor (WT).

Since the Segment Anything Model (SAM) [4], a second route has been available: a detector localizes the lesion with a bounding box and a general-purpose segmenter outlines it. Such segmenters are sensitive to how the box is placed [5]. The family runs from SAM 2 [6] to SAM 3 [7], with medical adaptation via low-rank tuning of a frozen encoder [8,9]; MedSAM [10] is its box-prompted member fitted on medical data at scale. A box is not the only possible prompt: BiomedParse [11] takes text, which removes the detector but not the need to locate the target, since the model must find it in the image itself. The detector-free comparator studied here is a medically pretrained model of this latter kind (Section 2.1).

The staged design is much smaller in memory and faster for whole tumors; by our estimate, it would be slower if all three regions were needed (Section S3), and it can fail in two places rather than one. The choice of design is usually decided by one number: the margin by which one beats the other on a test set. That number does not say which component failed. A pipeline may miss the tumor, or find it but outline it poorly; Dice adds the two together, so it cannot tell a practitioner whether to retrain the detector, replace the segmenter, or change the architecture. Prompting the segmenter with a box drawn from the ground truth separates the two. This oracle-box instrument is established [12,13], and it is the standard protocol for evaluating promptable segmenters on medical images [14]. The localize-then-segment design is established as well: MedLSAM [15] is the closest relative of the pipeline studied here, which pairs a real-time detection transformer (RT-DETR) [16] with a promptable segmenter. As far as we can establish, no study has used an oracle-box arm to split the gap between a pipeline and a detector-free model of a different design into terms that add up exactly.

The single number has two further weaknesses. It is usually measured on one cohort, yet few published medical imaging models are externally validated [17]. Those that are lose performance systematically [18,19,20], and a model can learn site-specific confounders rather than pathology [21]. Three cohorts are therefore used: a BraTS2020 subset held out by both models, the Río Hortega University Hospital glioblastoma collection (RHUH-GBM) [22], and BraTS-Africa [23]. They are ordered by provenance (same distribution, a different hospital, and a different continent), not by a measured distance; Section 2.1 compares them quantitatively. A range of shifts, rather than a single external cohort, tests whether the share of the loss carried by the detector stage depends on how far a cohort lies from the training data. The single number also usually comes from one training run, and single runs are unreliable [24,25,26,27,28]. When nnU-Net was trained with 50 seeds on brain tumor data, the best seed significantly beat many of the others [29]. Challenge rankings change with the aggregation method and with single test cases [30,31], and variance is rarely reported in medical image segmentation [32]. The main comparison is therefore repeated at a second detector seed and three adapter seeds (Section 3.5 and Section 3.6).

A last problem is specific to comparing two independently developed models on one public dataset. The detector and the adapter come from two earlier projects of the present authors, which split the same 368 BraTS2020 cases independently, so that 45 of the 55 test patients of one model were in the training set of the other. This training–test non-independence is a recognized leakage type [33] that inflates the reported accuracy in medical imaging [34,35] and propagates into meta-analyses [36]. The in-domain comparison therefore uses only the 24 patients held out by both models (Section S5).

**Aim and design:** This study asks where a box-prompted pipeline loses accuracy when it leaves its training distribution: in the detector that proposes the box, or after it. Four arms are run on every patient (Section 2.3): the deployable pipeline; an oracle-box arm that gives the same segmenter the ground-truth box; the detector-free model; and the detector-free model given the ground-truth box. The gap is the detector-free model’s Dice minus the pipeline’s. The difference between the oracle-box arm and the pipeline is the detector-stage term, and the remainder of the gap is a residual that mixes several factors and is not a measure of segmentation quality (Section 2.5). The analysis is methodological. It is performed on the whole tumor, where the decomposition can be measured in all three cohorts, and not on the regions that drive clinical decisions (Section 4). Section 2 describes the cohorts, the arms, and the decomposition; Section 3 reports the decomposition, the collapses, the robustness checks, and the sub-regions; and Section 4 discusses causes, practical use, and limitations.

**The contributions are as follows:**

1. **A decomposition of the gap.** The detector-stage term and the residual add up exactly to the gap between the pipeline and the detector-free model, so the detector’s share of a loss faced in deployment can be read directly. A fourth arm splits the residual into a prompt term and a model term (Section 2.4, Section 3.2 and Section 3.3);
2. **A result resolved on one external cohort.** On BraTS-Africa, the detector-stage term rises well above its in-domain value and accounts for most of the increase in the gap. On RHUH-GBM, a detector-stage cost is present, but its increase over the in-domain value is not resolved. No change in the residual is resolved on either cohort (Section 3.2), and on the tumor core, the pattern of the detector-stage term does not hold (Section 3.6);
3. **Single-box prompting is a limit for enhancing tumors, but not for the tumor core.** A single tight box often cannot tell an enhancing rim from the core it surrounds because both fit the same box [4,37], so the segmenter returns the core. The tumor core has no such limit: a correct box gives a clear headroom there, although a tumor-core detector trained here realized it only in part (Section 3.6);
4. **Evidence that out-of-domain single-run comparisons are unreliable.** On the tumor core, detectors that differ only in the training seed differ materially in the cohort Dice, and on BraTS-Africa, no detector seed resolves which system is better (Section 3.6).

## 2. Materials and Methods

### 2.1. Data

**Figure 1:**
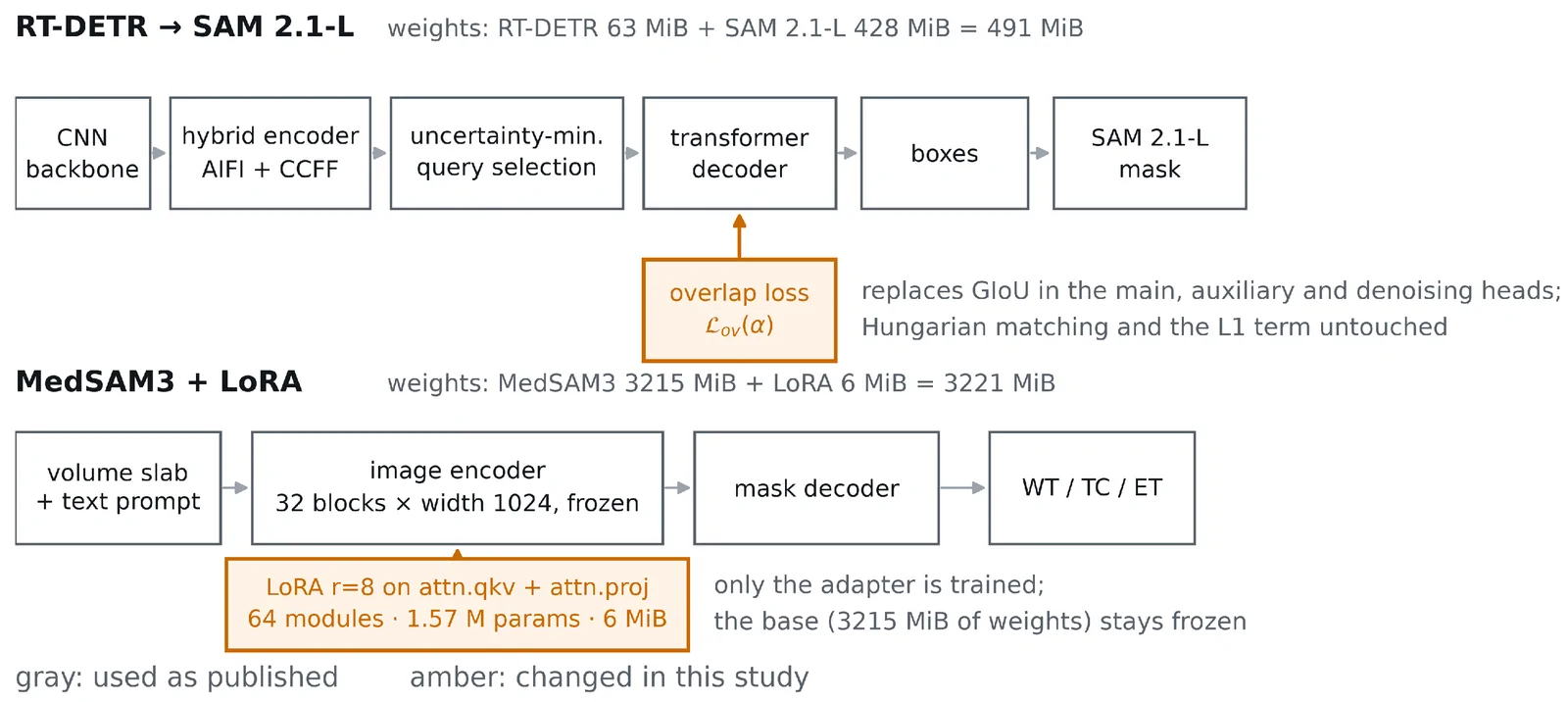
The two systems. Gray components are used as published; amber components were changed in this study: the overlap term in the detector’s box loss and the low-rank adaptation (LoRA) adapter on the frozen MedSAM3 encoder.

Figure 1 shows the two systems and marks the two components changed in this study. **BraTS2020** was split by patient for the detector study into 258 training, 55 validation, and 55 test cases (ratios of 0.7/0.15/0.15). The detector study and the adapter study are two earlier, unpublished projects of the present authors. Of the 369 released cases, 368 were used; the exclusion was made at ingest, before the split was drawn (Section S8).

**RHUH-GBM** (Río Hortega University Hospital, The Cancer Imaging Archive (TCIA), CC BY 4.0) provides 40 patients with three studies each. Only the preoperative study was used because the later studies contain resection cavities that neither model was trained on; one patient was excluded as off the BraTS grid (Section S8), leaving *n* = 39. **BraTS-Africa** (TCIA, BraTS 2023 release) contains 146 participants, 95 with glioma and 51 with other neoplasms. Only the **95 glioma** cases were used to limit the confounding of domain shift with tumor type; their diagnosis is presumed rather than pathologically confirmed (Table 1). The public challenge partition also numbers 95 cases, but the source papers do not state how the two sets relate, so every figure reported here is from the TCIA release.

Neither external cohort overlaps with BraTS2020, so both are held out from the task-specific training performed here (the detector and the LoRA adapter). The in-domain set is the 24 patients held out by both projects (Section S5); they were used to select both models, although not to train them, and Section 3.2 estimates the resulting bias. The detector-free arm runs the MedSAM3 of Jiang et al. [38], not the similarly named model of Liu et al. [39]. The configuration shipped with its repository lists BraTS MRI from the BiomedSegFM collection of the Computer Vision and Pattern Recognition (CVPR) 2025 challenge among its pretraining data, under BraTS 2023 sequence names. BraTS 2023 includes the BraTS2020 cases, so the held-out patients were likely included in the base model’s pretraining, and whether BraTS-Africa was included could not be established. This bears on the residual term only, not on the detector-stage term (Section 4.2).

Label values, grid geometry, and intensity were reconciled as set out in Section S8. Two preprocessing rules assume that the skull-stripped background is exactly zero; because RHUH-GBM is released z-score-normalized with a negative background, its background is instead derived from each volume (Section 3.2 and Section S8). The whole tumor, the union of every non-zero label under all three conventions, is unaffected by the label remapping, and this is the region on which the three-cohort result is measured. The narrower RHUH-GBM sub-region definition affects the tumor core and enhancing tumors only (Section 3.6 and Section 4.2).

**Cohort characteristics:** Table 1 lists what the source publications [3,22,23,40] report for each cohort and what can be measured from the released images. All released volumes are skull-stripped, co-registered, and resampled to 240 × 240 × 155 voxels at 1 mm isotropic spacing, so the native acquisition resolution cannot be recovered from the images. Intensities are released on different scales (non-negative with a zero background for the two BraTS cohorts, z-score-normalized with a negative background for RHUH-GBM), so intensities are not compared directly. Contrast is instead measured with an index that does not depend on intensity scale or offset. For each input channel (fluid-attenuated inversion recovery, FLAIR; contrast-enhanced T1-weighted, T1ce; T2-weighted, T2), this is the median intensity inside the whole-tumor reference minus the median in the normal-appearing brain, divided by the interquartile range of the normal-appearing brain. These measurements describe the cohorts; they are not used as causal explanations of the results (Section 4).

**Table 1:** Cohort characteristics.

	BraTS2020 Held-Out	RHUH-GBM	BraTS-Africa	*p*
patients	24	39	95	
origin (as reported)	19 contributing institutions [3]	one hospital, Spain; 11 of 40 patients first imaged at a second center [22]	six or seven imaging centers, Nigeria (sources differ) [23,40]	
field strength (as reported)	1.5 T and 3 T, not given per case [1]	1.5 T [22]	1.5 T [40]	
scanners (as reported)	varies by institution [3]	GE Signa HDxT; Philips Ingenia Ambition X; Philips Achieva [22]	Siemens Magnetom Essenza; Toshiba Vantage Elan; Philips Achieva; GE SIGNA Explorer; GE SIGNA Creator [40]	
acquired slice thickness (as reported)	not reported	FLAIR 1.2–4 mm by scanner [22]	3–5 mm by center [40]	
diagnosis (as reported)	high- and low-grade glioma (pre-2021 WHO grading), pathologically confirmed [1,2,3]	glioblastoma (WHO 2016), 38 de novo and 2 recurrent after treatment; 90% IDH wild-type [22]	presumed adult diffuse glioma, not pathologically confirmed [40]	
age and sex (as reported)	age per case, not summarized [3]	63 ± 9 years; 28 men, 12 women [22]	adults, not reported [40]	
reference segmentation (as reported)	manual, or manually revised after semi-automatic pre-segmentation; neuroradiologist-approved [1,2,3]	DeepMedic, corrected by two neurosurgeons [22]	nnU-Net pre-annotation, refined by expert review (sources differ) [23,40]	
intensity scale as released	non-negative, zero background	z-score, negative background	non-negative, zero background	
grade, patients used (released per case)	17 HGG, 7 LGG [3]	glioblastoma, WHO 2016 grade IV, in all 39 cases (the 4 IDH-mutant cases are astrocytoma, IDH-mutant, grade 4 under WHO 2021); 35 IDH wild-type, 4 IDH-mutant; 2 treated before the scan [22]	not released	
age in years and sex, patients used (released per case)	age 59.9 (55.8, 67.5), *n* = 12; sex not released [3]	age 64 (55, 69); 27 men, 12 women [22]	not released	
WT volume, cm^3^	99.8 (69.3, 147.9)	111.0 (58.3, 159.2)	162.9 (77.6, 232.1)	0.001
ET volume, cm^3^	14.7 (3.8, 23.5)	18.0 (8.5, 34.4)	31.1 (14.0, 64.6)	9.5 × 10^−4^
slices containing WT	68 (59, 77)	66 (54, 78)	78 (60, 88)	0.005
WT connected components	3.5 (2.0, 8.2)	1.0 (1.0, 2.0)	1.0 (1.0, 2.0)	5.4 × 10^−7^
contrast index, FLAIR	2.00 (1.44, 3.03)	2.15 (1.51, 2.63)	1.32 (0.94, 1.68)	8.5 × 10^−7^
contrast index, T1ce	−0.27 (−0.66, 0.02)	−0.37 (−0.58, −0.03)	0.21 (−0.02, 0.42)	1.8 × 10^−8^
contrast index, T2	1.38 (1.04, 1.92)	1.64 (1.30, 2.62)	1.74 (1.20, 2.36)	0.220

Reported rows are cohort-level statements from the source publications; measured rows were computed on the released images. Measured rows: median (interquartile range) over patients; *p*, Kruskal–Wallis test across the three cohorts, descriptive only. Pairwise comparisons with the held-out set and the remaining features are in Table S18 (Section S17.4). WHO, World Health Organization; IDH, isocitrate dehydrogenase; WT, whole tumor; ET, enhancing tumor. The BraTS-Africa sources differ on the number of centers and on the review process. Rows marked “released per case” summarize the per-patient files distributed with each dataset for the patients used here (median and interquartile range; Section S17.4); scanner and sequence parameters are not available per case for any cohort. Connected components are counted in three dimensions (26-connectivity) on the whole-tumor reference. Scanner manufacturers: GE HealthCare (Chicago, IL, USA); Philips Healthcare (Best, The Netherlands); Siemens Healthineers (Erlangen, Germany); Toshiba Medical Systems (now Canon Medical Systems, Otawara, Japan).

### 2.2. The Detector’s Training Objective

The detector is RT-DETR, trained with an asymmetric overlap term in its box loss. For a matched prediction *P* and target *G*, this term is *α*(1 − IoU) + (1 − *α*)(1 − *C_p_*), where IoU is the intersection over union, and *C_p_* = |*P* ∩ *G*|/|*P*| is the fraction of the predicted box that lies on the target; *α* is a mixing weight, not the exponent of α-IoU [41]. This term, written as *L_ov_*, replaces the generalized IoU (GIoU) component [42] of the post-matching box loss in the main, auxiliary, and denoising heads; Hungarian matching and the L1 term are unchanged. It belongs to an established family of IoU variants that add a geometric penalty [42] or a power [41], and of asymmetric indices that weight the two set differences separately [43,44,45,46]; it is part of the set-up, not a contribution. The detector configuration is in Table S11, and the adapter configuration is in Table S12. The detector used here is run 37, selected from 37 training runs; Section S10 estimates what that selection is worth. The confidence threshold of 0.55 was fixed in the detector study before its test split was scored. It is not the F1 optimum of the validation split (0.46) but lies inside the flat band (0.45–0.65) where F1 is within about 0.005 of that peak, and it was not tuned again here (Section S4). This keeps test data out of the choice, although such tuning is known to inflate results only modestly [47]. Section 3.4 shows how the results change with the threshold. Volumetric Dice is pooled over a patient’s voxels (Section 2.4 and Section S14) and cannot be read against a BraTS leaderboard row (Section 4.2).

### 2.3. Arms

**Figure 2:**
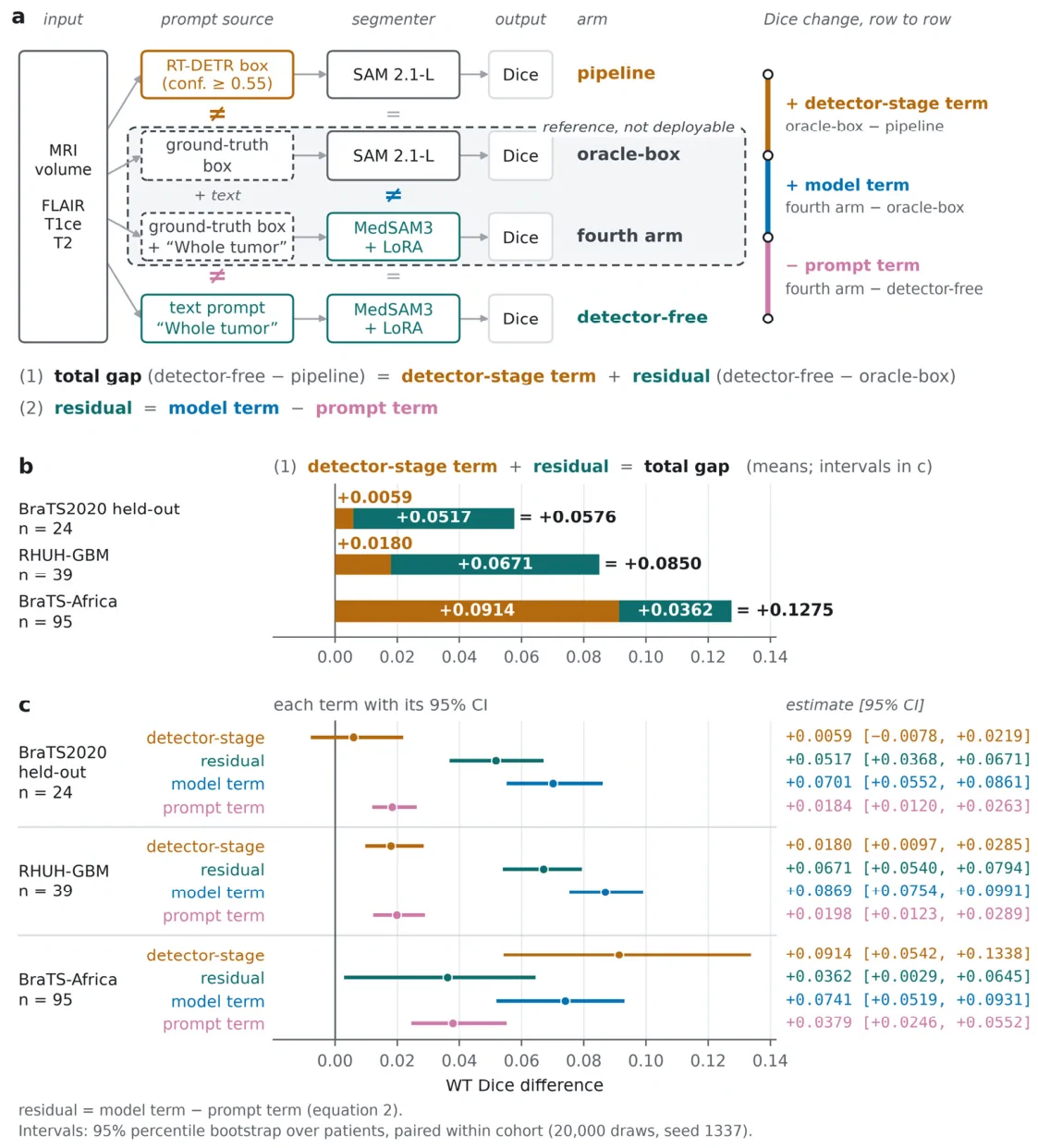
The four arms and the decomposition. (**a**) Neighboring arms differ in one component of the schematic (≠; Table 2 lists every factor that changes); the oracle-box and fourth arms use the ground-truth box and are not deployable. (**b**) The detector-stage term plus the residual equals the total gap in each cohort. (**c**) Each term with its within-cohort 95% bootstrap confidence interval; the contrasts between cohorts are in Section 3.2.

Four arms are run for every patient (Figure 2a):

- The **pipeline** passes RT-DETR boxes (run 37, confidence ≥ 0.55) to SAM 2.1-L;
- The **oracle-box** arm passes the tight bounding box of the ground-truth region to the same segmenter. It needs the answer, so it is not deployable and is used only as a reference;
- The **detector-free** arm is MedSAM3 with a LoRA adapter, prompted with a region name and no box;
- The **fourth arm** gives the same detector-free model the ground-truth box in addition to the region name. It is used only to split the residual (Section 2.4).

**Table 2:** Factors that change across each term.

Factor	Detector Stage (Oracle-Box − Pipeline)	Model (Fourth Arm − Oracle-Box)	Prompt (Fourth Arm − Detector-Free)	Residual (Detector-Free − Oracle-Box)
Source of the box (ground truth or detector)	Changes	—	—	—
Knowledge of which slices contain a tumor	Changes	—	Changes	Changes
Boxes per slice on a fragmented target	Changes (Section S7)	—	—	—
Box prompt (absent, or added to the region name)	—	—	Changes	Changes
Region name given with the box	—	Changes	—	—
Adapter used with a box prompt (training prompt mode not recorded)	—	Changes	Changes	—
Segmenter architecture and size	—	Changes	—	Changes
pretraining data	—	Changes	—	Changes
Task-specific adapter (LoRA)	—	Changes	—	Changes
Input normalization and resizing	—	Changes	—	Changes

Terms of Equations (1) and (2). A dash means the factor is held fixed across that term. The detector-stage term changes only the prompts (where the boxes are, on which slices, and how many), never the segmenter; the residual changes six factors at once.

All arms see the same volumes and slices, normalized as each model requires (Section S14), and are scored by the same code. On RHUH-GBM, this required correcting the normalization of every arm, including the adapter’s own (Section 3.2 and Section S8); the model’s published preprocessing is reported as a sensitivity analysis (Table S10). SAM 1 with a base Vision Transformer encoder (ViT-B), the segmenter of the original detector study, is used as a second box-prompted segmenter (Section 3.5); SAM 2.1-L denotes the large variant of SAM 2.1. An identical protocol (the same detector, threshold, segmenters, and adapter, and every axial slice) was applied to all three cohorts. All code was written in Python 3.14 (Python Software Foundation, Wilmington, DE, USA) with PyTorch 2.11 (PyTorch Foundation, San Francisco, CA, USA), and inference ran on an NVIDIA GeForce RTX 5060 Laptop GPU (NVIDIA, Santa Clara, CA, USA).

The oracle-box arm is an instrument, not a method. It receives the tight box on every slice that contains the region, so it knows both where the tumor is and which slices contain it. It therefore shows what *perfect localization under this prompt protocol* delivers, not what any detector could reach: it is a reference rather than a ceiling, and the pipeline sometimes scores above it (Section 3.2). When the target has several components on a slice, the pipeline may emit several boxes while the oracle-box arm receives one; Section S7 reports the oracle-box arm under one box per component as well.

### 2.4. The Decomposition

Let *d_orc_*, *d_pipe_*, *d_free_*, and *d_box_* be the volumetric Dice of the oracle-box, pipeline, detector-free, and fourth arms for one patient. The detector-free arm’s advantage over the pipeline decomposes exactly as in Equation (1):

*d_free_* − *d_pipe_* [total] = (*d_orc_* − *d_pipe_*) [detector stage] + (*d_free_* − *d_orc_*) [residual]. (1)

The fourth arm splits the residual in turn, as in Equation (2):

(*d_free_* − *d_orc_*) [residual] = (*d_box_* − *d_orc_*) [model] − (*d_box_* − *d_free_*) [prompt]. (2)

Both are identities: they hold for every patient and therefore for every cohort mean. Figure 2 shows the four arms, the component that differs between each pair, and the measured terms.

Volumetric Dice for an arm with per-slice predictions *{A_z_}* against reference *{B_z_}* over the evaluated slice set *Z* is computed on pooled voxels rather than averaged per slice, so that slices carrying a few tumor voxels do not weigh as much as slices through the center of the tumor, as in Equation (3):

Dice = (2Σ*_z__∈Z_*|*A_z_* ∩ *B_z_*|)/(Σ*_z__∈Z_*|*A_z_*| + Σ*_z__∈Z_*|*B_z_*|). (3)

### 2.5. What the Two Terms Measure

**The detector-stage term** is the cost of using the detector’s boxes instead of ground-truth boxes, with the segmenter and the prompt type held fixed. In the title and the figures, a detector-stage failure is a loss the oracle-box arm does not share: at the cohort level, this is the detector-stage term; at the patient level, this is a collapse. It belongs to this prompt protocol (one tight box per slice, SAM 2.1-L) and is not a measure of localization accuracy alone. It includes slices on which the detector emits no box, boxes on the wrong structure, and correct boxes of a different shape; Section S6 separates the first of these from the other two. It is an average over patients and is distinct from a **collapse**, defined here as a patient on whom an arm scores below 0.5 Dice. Collapses contribute to the term, heavily on BraTS-Africa (Section 3.2 and Section 3.4), but a positive term does not require collapses, and the term is reported with and without them.

**The residual** compares two different systems on the same patients. It is not a controlled measure of segmentation ability, because several factors change across it at once (Table 2). The fourth arm gives the detector-free model the same box as the oracle-box arm, together with the region name it always receives. Its prompt term therefore measures what adding a box gains this model, not a swap of prompt type, and its model term still changes architecture, pretraining, adapter, and input processing together, so no ordering of the two model families is drawn from it (Section S9). The comparison as a whole is a comparison of two systems on the same patients, not of one architecture against another.

### 2.6. Statistics

**Endpoint and primary contrasts:** The endpoint is volumetric whole-tumor Dice. Two contrasts are primary: the detector-stage term on each external cohort minus the same term in the domain (BraTS-Africa − held-out; RHUH-GBM − held-out). They follow from the design but were not pre-registered. On RHUH-GBM, the contrast was re-estimated after a preprocessing correction made once the results had been seen (Section 3.2). All other analyses are exploratory.

**Bootstrap intervals:** Unless stated otherwise, every confidence interval (CI) is a percentile bootstrap with 20,000 resamples, built as follows. (i) Resampling is by patient: a resample draws *n* patients with replacement from a cohort of *n*. (ii) Within a cohort, every patient passes through every arm, so the arms of a patient stay together. Each resampled patient contributes its own per-patient difference (for example, oracle-box minus pipeline), and the statistic is the mean of the resampled differences. (iii) Between cohorts, the patients are different people, so each cohort is resampled independently, and the statistic is the difference of the two resampled means. (iv) The 2.5th and 97.5th percentiles of the 20,000 statistics form the interval. This is an interval on the mean difference, as recommended for segmentation [48], not the spread of individual per-patient differences, which is much wider. The departures are stated where they occur: Table S13 uses 10,000 resamples at generator seed 0; Sections S6 and S17.5 give intervals on ratios; proportions in Section 3.4 and Section S17.8 carry exact binomial (Clopper–Pearson) intervals; and the threshold sweep of Table S15 draws its own resamples, so its intervals at 0.55 differ from those of Section 3.2 in the fourth decimal. All intervals are conditional on the trained detector and adapter; seed variation is examined separately (Section 3.5 and Section 3.6). Bias-corrected and accelerated (BCa) intervals, which allow for the skew of the per-patient terms, lead to the same conclusions for the within-cohort terms (Section S11) and for both primary contrasts ([+0.0492, +0.1353] and [−0.0077, +0.0281]). Within-cohort comparisons are also tested with the Wilcoxon signed-rank test.

**Multiplicity:** The two primary contrasts are tested with a Holm correction over the pair, using two-sided bootstrap *p*-values from the same resamples as their intervals (obtained by inverting the resampling distribution, not by resampling under the null), and Bonferroni-adjusted 97.5% intervals are reported beside the 95% intervals (Section 3.2). A Mann–Whitney test on the per-patient terms gives a rank-based check. Exploratory results are not adjusted.

**Effect sizes and robustness:** The per-patient terms are right-skewed, so means are reported together with the median, the 20% trimmed mean, and the Hodges–Lehmann estimator. Within a cohort, the matched-pairs rank-biserial correlation is reported as a standardized effect size. Between cohorts, Cliff’s delta gives the probability that a patient of one cohort has the larger term, minus the probability of the reverse. Intervals for these estimators use the same resampling scheme (Section S17).

**Precision of the in-domain set:** The in-domain set has 24 patients. Its precision is described by the width of the intervals and by a leave-one-patient-out analysis (Section 3.2 and Section S17.6). The bootstrap reflects sampling variation only; it cannot remove the bias that comes from these patients having been used in model selection (Section 3.2).

**Empty regions and boundary metrics:** Patients whose reference contains no tissue in a region are excluded from that region’s mean and reported separately (Section S8). Boundary agreement is measured on the reconstructed volume by the 95th percentile Hausdorff distance (HD95) and the normalized surface Dice (NSD) at a 2 mm tolerance. NSD is computed on boundary voxels as an approximation to the area-weighted definition (Section S2), with the same code for every arm. An empty prediction has no surface and is counted separately rather than scored (Section S2).

### 2.7. Use of Generative AI

The authors used Claude Code (version 2.1; Anthropic, San Francisco, CA, USA) as an assistive tool for programming support, language editing, and consistency checks. All scientific content, including the study design, the analyses, and their interpretation, is the authors’ own; the authors reviewed all AI-assisted output and take full responsibility for this publication.

## 3. Results

### 3.1. Overall Performance

Table 3 gives the three arms for each cohort, from detector run 37 with seed 1337 and LoRA adapter seed 42. The detector-free arm leads in overlap in every cohort, with 22 of 24, 38 of 39, and 95 of 95 patients, and the gap is larger in both external cohorts than in the domain.

The boundary metrics show that the two systems fail differently. The detector-free arm has the highest NSD of the three arms in every cohort, but its HD95 has a heavy tail: it exceeds 20 mm in 7/24, 13/39, and 37/95 patients, against 1/24, 4/39, and 20/92 for the pipeline. In every one of those detector-free cases, the prediction contains at least one component that does not touch the reference. These detached components are small, with medians of 2.4%, 2.2%, and 4.6% of the predicted volume in those patients, so they barely change Dice, but they dominate a distance metric. Keeping only the largest connected component of each detector-free prediction, a standard post-processing step applied to no arm in the main analysis, removes most of this tail: the median HD95 becomes 3.30, 2.00, and 4.00 mm and exceeds 20 mm in 1/24, 1/39, and 9/95 patients, while its Dice barely moves (0.9179, 0.9385, and 0.8899). Applied to the pipeline, the same filter lowers its BraTS-Africa Dice to 0.7283, because the components it removes contain true tumors (Section S17.2). The pipeline’s boundary errors come instead from missed tumors (Section 3.4 and Section S6).

### 3.2. The Decomposition and the Primary Contrasts

Table 4 splits the gap in Table 3 into the detector-stage term and the residual (Figure 2b,c).

**On BraTS-Africa, the detector stage carries the increase.** The detector-stage term rises from +0.0059 in the domain to +0.0914 on BraTS-Africa, and this contrast survives the Holm correction and every robust estimator (Table 5). In that cohort, the term is 72% of the gap (95% CI of 47% to 98%), so it is the larger share, although it is not demonstrably more than half. Its point estimate exceeds the increase in the gap over the in-domain value (122%, 77% to 193%) because the residual is slightly smaller there than in the domain; the interval supports most of the increase, not all of it, and the residual contrast (Table 4) still admits a small residual increase (Section S17.5).

**On RHUH-GBM, the increase is not statistically resolved.** Here, the detector does cost something: +0.0180 [+0.0097, +0.0285], with the interval excluding zero. The increase over the in-domain value, however, is +0.0120 [−0.0062, +0.0295], and it is not distinguishable from zero under any estimator (Table 5). The increase in the gap on RHUH-GBM is split between the detector-stage term (+0.0120) and the residual (+0.0154), and neither is resolved. This is a failure to detect an increase, not evidence that there is none: increases of up to +0.0295 (+0.0320 under the Bonferroni interval) are compatible with the data.

**The term is concentrated in a few patients:** The per-patient term is right-skewed; the top five of the 95 BraTS-Africa patients carry 47% of it, and the nine collapses carry 60%. The direction of the BraTS-Africa contrast, and its interval excluding zero, survive the median, the 20% trimmed mean, and the removal of the collapses. Its size does not: for the median patient, finding the box costs 0.005, 0.010, and 0.016 Dice in the three cohorts (Table S19; Section S11). Within cohorts, the matched-pairs rank-biserial correlation of the term is 0.21 in the domain, 0.75 on RHUH-GBM, and 0.68 on BraTS-Africa (Section S17.5).

**No change in the residual is resolved:** It increases on RHUH-GBM (+0.0154) and falls on BraTS-Africa (−0.0155), and neither contrast excludes zero (Table 4). On the whole tumor, the term whose growth outside the training distribution is resolved is therefore the detector-stage term, and it is resolved on BraTS-Africa only.

**The two external cohorts differ in the mean but not clearly in the typical patient:** In the exploratory contrast between the two external cohorts, the mean detector-stage term is larger on BraTS-Africa by +0.0734 [+0.0351, +0.1162], but the rank-based estimators do not separate them (Cliff’s δ +0.17 [−0.03, +0.36] in Table 5; Hodges–Lehmann shift +0.0074 [−0.0013, +0.0178] in Section S17.5). The difference between the two cohorts lies mainly in the BraTS-Africa tail of collapses, so the result is stated cohort by cohort rather than as a difference between them.

**The in-domain reference is small and was used in model selection:** The 24 held-out patients were not used in the task-specific training of either model, but they were used to select both. The detector’s own 55-patient split was not used to choose the threshold, but it was used to choose run 37 (Section S10 shows that this choice is worth +0.0009 Dice). Both biases raise the in-domain pipeline score, which shrinks the in-domain term and so favors the claim made here. Against the cleanest in-domain reference available, the BraTS-Africa contrast narrows but still excludes zero, while the RHUH-GBM contrast falls to +0.0026, spanning zero (Section S5). No single held-out patient drives the result: removing each in turn leaves the BraTS-Africa contrast between +0.0818 and +0.0913 and the RHUH-GBM contrast between +0.0085 and +0.0179 (Section S17.6).

**A preprocessing fault in one cohort, and what correcting it changed:** Two of this study’s own rules assumed that the skull-stripped background is exactly zero, which is not true of RHUH-GBM (it is released z-score-normalized, with a negative background). The first rule discarded a median 43% to 62% of the brain across the three input channels, and the second rule cost usable slices in 33 of the 39 patients; both were corrected to derive the background from the volume (Section S8). The correction moved the two box arms in opposite directions, consistent with the fault having made the oracle-box arm’s task easier and the detector’s task harder. The detector-stage term fell from +0.0692 to +0.0180, and its contrast against the held-out set fell from +0.0633 to +0.0120, which spans zero. The detector-free arm carried the same flaw through the adapter’s own normalization and was re-run as well, rising by +0.0806. Table S17 gives every arm before and after the correction. The corrected run is primary for three reasons. All arms then see the same volumes, the background is treated as in the other two cohorts, and an artefact of the mask is not mistaken for a property of the cohort. The model’s published preprocessing is reported as a sensitivity analysis (Table S10). Two RHUH-GBM patients had been treated before their preoperative scan; without them, the term is +0.0156 [+0.0086, +0.0250], and its contrast against the held-out set is +0.0097 [−0.0080, +0.0262] (Section S17.3).

**The oracle-box arm is a reference, not an upper bound:** For 41 of the 158 patients (11 of 24, 9 of 39, and 21 of 95), the pipeline scores above it. This is consistent with the sensitivity of promptable segmenters to box geometry [5]. The oracle-box arm leads in every cohort mean, so the decomposition is unaffected. On BraTS-Africa, the term grows because the pipeline degrades: the pipeline arm falls by 0.1059 with an interval excluding zero, while the oracle-box arm’s own movement, −0.0205 [−0.0476, +0.0088], is not resolved (Table S6). On RHUH-GBM, neither arm moves detectably.

**There is no evidence that lesion size explains the difference between cohorts.** Within each cohort, smaller tumors carry a larger term where there is an association at all, as the small tumors of the collapses also show (Section S17.8), but the external cohorts have *larger* tumors (Table 1, Section S11). Lesion size therefore cannot account for the difference between cohorts, although other differences between them remain possible (Section 4.2).

**Prompt protocol:** Under one box per connected component (Section S7), every detector-stage term increases, including the in-domain term (+0.0310, interval excluding zero). The BraTS-Africa contrast is almost unchanged, and the RHUH-GBM contrast becomes smaller, so both cohort-level conclusions hold under either protocol.

### 3.3. The Fourth Arm Splits the Residual

Table 6 reports the prompt term and the model term of Equation (2) on all three cohorts, at adapter seed 42; the arms they are differenced against are in Table 3.

All six terms exclude zero, and the two behave differently between cohorts. The prompt term grows with external shift on BraTS-Africa (+0.0195 against the held-out set, excluding zero), but not on RHUH-GBM (+0.0014), which is the same pattern as the detector-stage term: what a box adds to this model is not a fixed amount. Neither contrast of the model term excludes zero (+0.0168 [−0.0028, +0.0363] and +0.0040 [−0.0231, +0.0283]). This is a failure to reject, not a demonstration of equivalence; the first interval admits a contrast as large as the prompt term’s on BraTS-Africa. On BraTS-Africa, this pattern is consistent with locating the target being what degrades out of domain, whether by box or by prompt; the contrast is exploratory and unadjusted, so this is a hypothesis rather than a finding.Both terms also carry the oracle’s knowledge of which slices contain a tumor (Table 2). Restricting the pipeline and the detector-free arm to the slices that contain tumors measures that share: it is +0.0006, +0.0020, and +0.0034 for the detector-stage term, and +0.0068, +0.0080, and +0.0119 for the prompt term; the rest of the prompt term, which is what the box adds within a tumor-bearing slice, is +0.0259 [+0.0156, +0.0403] on BraTS-Africa (Section S17.9).No degradation could be resolved in the model term. This term, however, combines the segmenter with its pretraining, adapter, and input processing, so it is not a measure of segmentation ability and does not order the two models (Table 2, Section S9).

### 3.4. Collapses and the Confidence Threshold

Nine of the 95 BraTS-Africa patients score below 0.5 volumetric Dice through the pipeline, and 2 score below 0.5 through the detector-free arm; no patient in either of the other cohorts scores below 0.5 through any arm. The oracle-box arm collapses on none of the 158 patients, which puts a one-sided 95% upper bound of roughly 1.9% on its collapse rate. Because it differs from the pipeline in the prompts it receives and not in the segmenter, every pipeline collapse is attributable to the detector stage. On BraTS-Africa, this is mostly a matter of missing boxes: slices on which the detector proposes nothing account for 82% of the term there when they are substituted first (Section S6).

**The confidence threshold:** The deployed threshold of 0.55 was inherited from the detector study (Section S4). Re-running the pipeline at 0.45, 0.35, 0.25, and 0.15 on all three cohorts shows that lowering it helps only on BraTS-Africa (Figure S3, Table S15). There, the term falls from +0.0914 to a minimum of +0.0661 [+0.0393, +0.0975] at 0.25, and the collapses fall from nine to four, as recall rises from 0.720 to 0.767 and precision falls from 0.898 to 0.778. At 0.15, the extra boxes are increasingly incorrect, and the term rises again. In the other two cohorts, every lower threshold makes the term larger, reaching +0.0238 in the domain and +0.0279 on RHUH-GBM at 0.15; in the domain, it produces a collapse that 0.55 does not. The best BraTS-Africa threshold was chosen based on that cohort’s own test data, so it is an exploratory upper bound, not a deployable setting. Even so, it recovers about a quarter of the term, and no threshold brings the BraTS-Africa term close to its in-domain value.

**What the collapses have in common:** A diagnostic that uses the reference (no box on a majority of the tumor-bearing slices) fires on seven of the nine collapses. These include the three on which the detector emits no box at all, where the volumetric Dice is exactly 0.000. It also fires on two patients at 0.504 and 0.593 Dice. Because it needs the reference, it cannot be deployed. A check that uses only the detector’s output flags a patient when no 27 consecutive slices carry a box, with 27 being the shortest such run in the held-out set, so that no held-out patient is flagged. Its form was chosen after the collapses had been seen; only the cut-off came from the held-out set. On BraTS-Africa, it flags 17/95 patients and catches 8 of 9 collapses (Clopper–Pearson 95% CI: 52% to 99.7%), so 8/17 flags (47%, 23% to 72%) are collapses; the false-alarm rate is 9/86 (10.5%, 4.9% to 18.9%). The false alarms have small tumors (median whole-tumor volume of 65.4 versus 164.9 cm^3^), as a run length counted in slices implies. The check raises no flag on RHUH-GBM, which has no collapses (Section S17.8). Compared with the other 86 patients, the collapses had lower FLAIR contrast (median index of 0.71 versus 1.34), a smaller brain volume (1407 versus 1559 cm^3^), and tumors lower in the brain (relative axial position of 0.30 versus 0.57). In 4 of 9 versus 17 of 86, the brain mask reaches the edge of the volume, and one (BraTS_SSA_00129) has a brain volume of only 878 cm^3^, consistent with a truncated field of view. On their tumor-bearing slices, the detector’s highest score was a median of 0.18 versus 0.94, so these are not near-misses of the threshold. An automatic rule (most tumor voxels in the lowest 30% of the brain and within 25 mm of the midline) places the tumor low and near the midline, which suggests an infratentorial location, in 4 of 9 collapses versus 4 of the 86 other patients (Fisher *p* = 0.002), and in 0 of the 258 patients in the detector’s training split. The rule is crude and was not checked by a radiologist. These comparisons are descriptive (Section S17.8).

**Figure 3:**
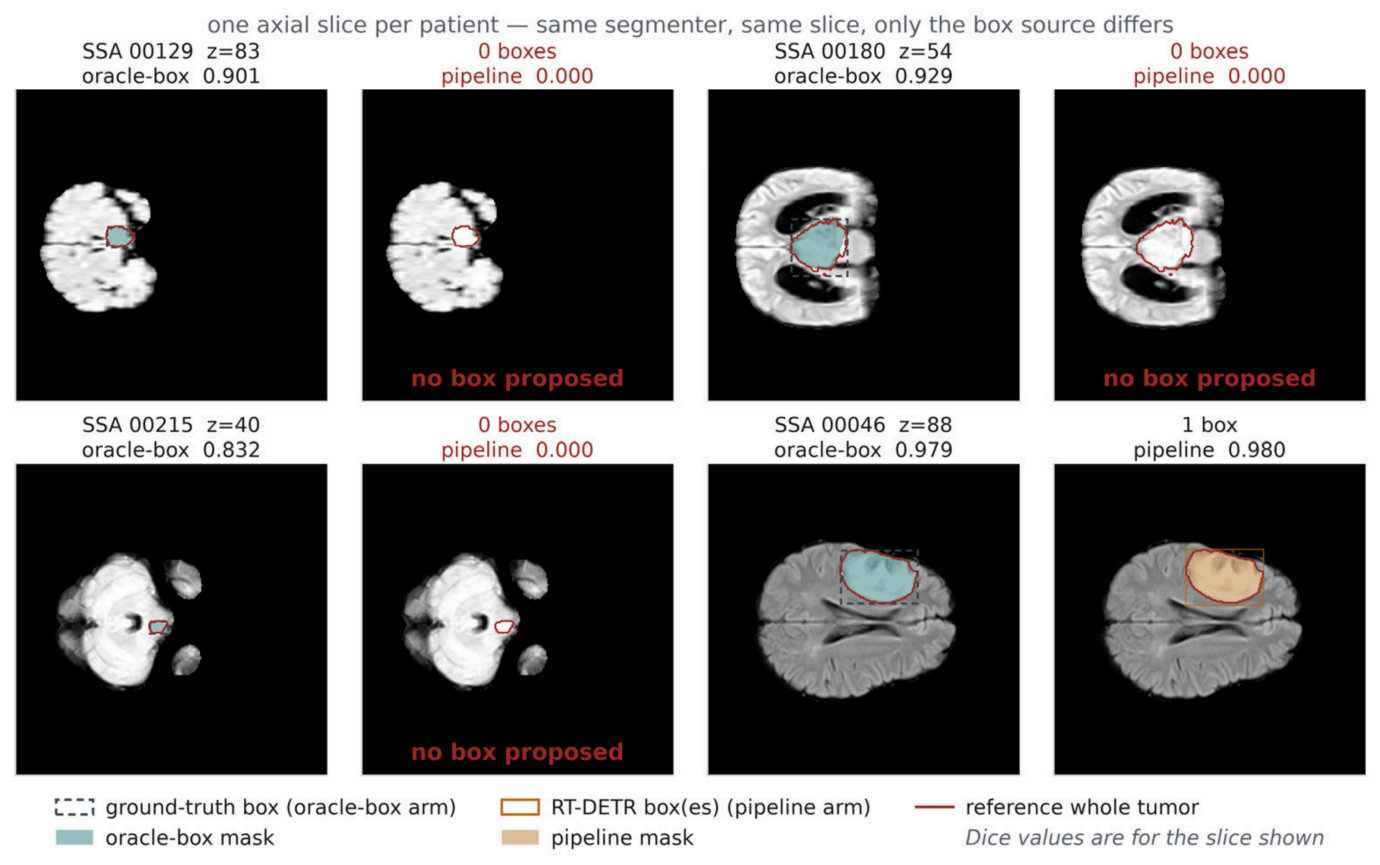
Detector-stage failures on BraTS-Africa. One axial slice per patient, with the same segmenter in both panels of a pair, so that only the source of the box differs. In the first three patients, the detector proposes no box; in the fourth, it costs nothing. Images: BraTS-Africa collection, The Cancer Imaging Archive (https://doi.org/10.7937/v8h6-8x67 (accessed on 10 October 2026)) [40], used under the CC BY 4.0 license; the boxes, masks, and outlines were added in this study.

**The other arm also collapses on the same patients:** The detector-free arm collapses on two BraTS-Africa patients at all three adapter seeds, and the pipeline collapses on both. The detector-free arm collapses on no patient that the pipeline handles, and on neither of the other cohorts. Given the same ground-truth box, MedSAM3 still scores 0.231 on BraTS_SSA_00215, one of its two collapses, where SAM 2.1-L reaches 0.858 with that box.Figure 3 shows three of the nine pipeline collapses, and the one patient for which the detector costs nothing. For the first three, the detector proposes no box, and the volumetric Dice is exactly 0.000, while the oracle-box arm reaches 0.832 to 0.929 on those slices. All ten patients in this series are shown in Figure S1 (Section S6).

Excluding the collapses does not remove the effect. Over the remaining 86 BraTS-Africa patients, the gap is +0.1047 (detector-free arm ahead on 86 of 86; Wilcoxon *p* = 8.0 × 10^−16^), and the detector-stage term falls from +0.0914 to +0.0406. Its contrast against the held-out set still excludes zero, at +0.0346 [+0.0112, +0.0582].

### 3.5. Robustness to the Segmenter and to Seeds

Two sensitivity checks re-measure the contrast—a second segmenter (SAM 1 ViT-B) and a second detector seed—and it survives both. Under both, the in-domain interval spans zero, the external within-cohort intervals exclude it, and only the BraTS-Africa contrast excludes zero. The second detector seed leaves the term at +0.0060, +0.0164, and +0.0755. The two segmenters are of the same family but differ in architecture, size, and training data, so the term is robust to a second SAM-family segmenter; whether it would hold for a segmenter of another kind was not tested. A third check varies the detector-free arm across three LoRA adapter seeds; this leaves the detector-stage term untouched and keeps the total gap excluding zero everywhere, with spreads of 0.0038, 0.0061, and 0.0017 (Section S12, Tables S7–S9).

### 3.6. Sub-Regions and the Enhancing-Tumor Limit

The oracle-box arm was also scored on the tumor core and enhancing tumors, against the detector-free arm on the same regions (Table 7).

**Figure 4:**
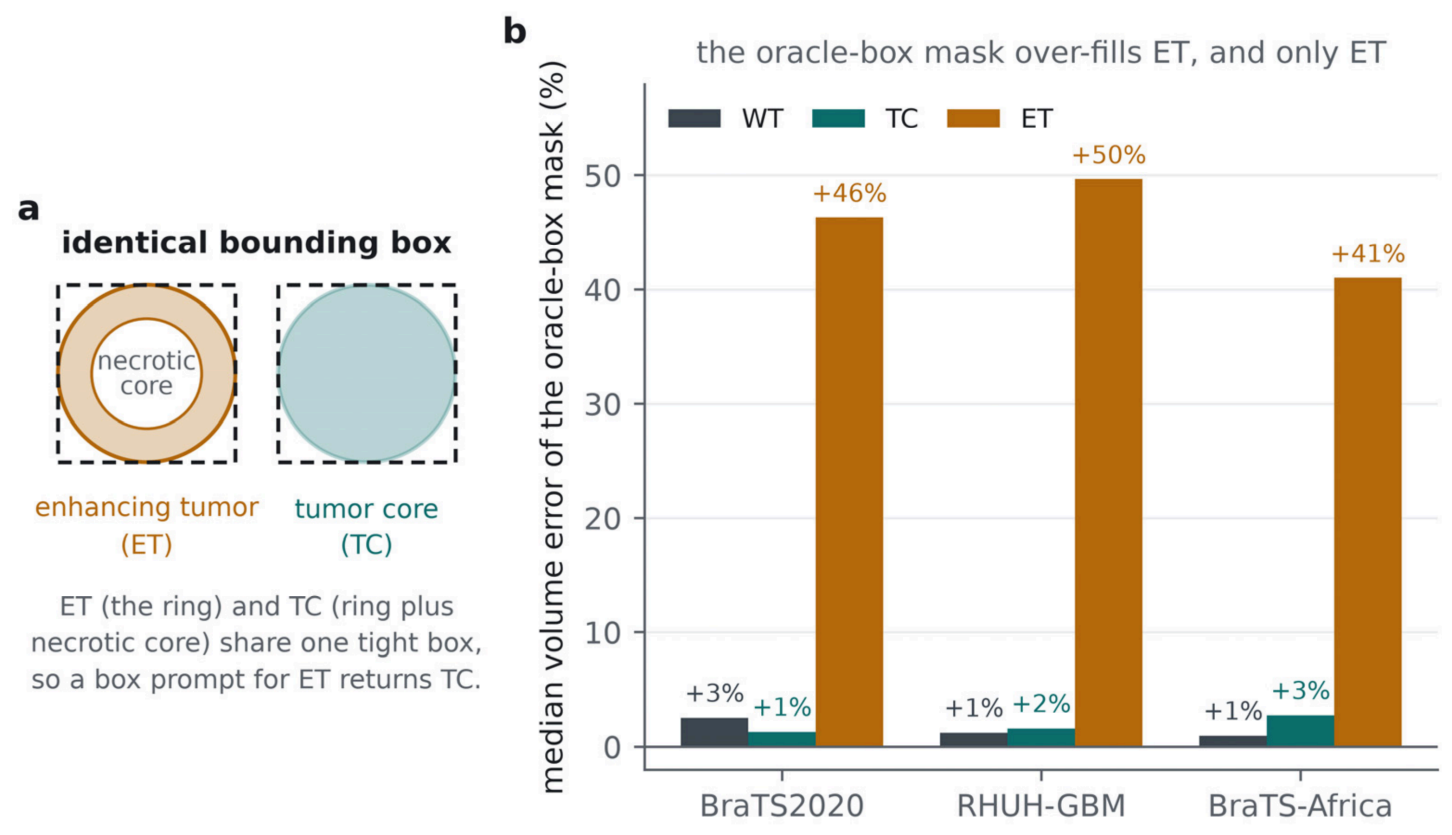
Why a single box cannot separate an enhancing tumor from the core. (**a**) The two share one tight box whenever the enhancing tissue reaches the core’s outermost voxel on every side, as a rim around a necrotic center does; the enhancing mask fully encloses the non-enhancing core on only 3.0%, 51.6%, and 32.7% of enhancing slices (held-out set, RHUH-GBM, and BraTS-Africa), and most of the other coincidences are bulky enhancing masses that no non-enhancing tissue extends beyond. The two boxes are identical on 48.1%, 99.4%, and 85.2% of the slices carrying both regions. (**b**) Median volume error of the oracle-box mask by region and cohort.

**An enhancing tumor is limited by the prompt** (Figure 4): Under a single tight box, an enhancing tumor and the core it lies in share the same box whenever the enhancing tissue reaches the core’s extreme voxel in every direction, as a rim around a necrotic center does. The two tight boxes are identical on 48.1%, 99.4%, and 85.2% of the slices carrying both regions in the three cohorts. Where they coincide, the enhancing-tumor prompt returns the core: the oracle-box mask over-fills the enhancing tumor by a median of 41% to 50% in every cohort, while the whole tumor and the tumor core remain within a few percent. A more accurate detector trained to the same tight-box target cannot remove this because the target itself is ambiguous; the detailed geometry is in Section S17.7.

**The tumor core is not limited in this way.** Its oracle-box score equals or exceeds that of the whole tumor in every cohort and stands above the detector-free model at every adapter seed (Table 7), so a correct box gives the box-prompted design clear headroom on the tumor core. This headroom is an oracle quantity: the oracle-box arm also knows which slices contain the region. For an enhancing tumor, the headroom changes sign across cohorts and, in the domain, across adapter seeds; it is positive at every seed only on BraTS-Africa, where it remains an oracle quantity. No enhancing-tumor detector was trained, so no advantage of either deployable design is established there; the case for detector-free models in that region rests on the limit of the single box, not on a measured difference. What the tumor core lacks is a trained detector. The same architecture trained on tumor-core boxes at three seeds recovers 30% to 43%, 90% to 92%, and −25% to 12% of the headroom on the three cohorts (Section S16, Table S13); the resulting advantage over the detector-free model is resolved only on RHUH-GBM, whose core label is narrower (Section S8). On the tumor core, the detector-stage term across those seeds is +0.0750 to +0.0930 in the domain, +0.0122 to +0.0156 on RHUH-GBM, and +0.1373 to +0.1951 on BraTS-Africa (Table S14). Two whole-tumor findings therefore do not hold in this region: the in-domain term excludes zero at every seed, and RHUH-GBM falls below the held-out set rather than above it.

**Seed instability affects both designs:** On BraTS-Africa, the three tumor-core detectors differ by only 0.0098 in run detection F1, which is seed noise, yet their pipeline Dice spans 0.0578. Against the detector-free model’s 0.6956, one seed falls below, one is within 0.001, and one is above, so these runs do not settle which system is better. The detector-free model at three LoRA seeds varies too: its tumor-core ranges are 0.0090, 0.0261, and 0.0188 against the pipeline’s 0.0180, 0.0033, and 0.0578, so with three seeds, neither design can be called the noisier one (Figure S2). On the whole tumor, by contrast, the second detector seed moves the term by 0.016 at most, which is far less than the gap (Section 3.5).

## 4. Discussion

On the whole tumor, the increase in this box-prompted pipeline’s gap out of the domain was resolved as a detector-stage effect on BraTS-Africa and was not resolved on RHUH-GBM.. On BraTS-Africa, the detector stage accounts for most of the increase in the gap: its term rises from +0.006 to +0.091 Dice. The contrast survives a multiplicity correction, the robust estimators, a second detector seed, and a second segmenter, and no confidence threshold brings the term near its in-domain value. On RHUH-GBM, the detector also costs something, but no increase over the in-domain value was resolved, and increases of up to about 0.03 Dice remain compatible with the data. No change in the residual was resolved in either cohort. Whether the two external cohorts differ from each other was tested only as an exploratory contrast, which separates their means but not their typical patients (Section 3.2). The wider lesson concerns measurement rather than these two systems. Under an identical protocol, the end-to-end gap varies by more than a factor of two across the three cohorts (Table 3), but by almost nothing across adapter seeds (Section 3.5), as the external-validation literature predicts [17,18,19,20,21]. Only a decomposition shows which component is responsible.

That is what makes the result actionable. The oracle-box arm is an established instrument [12,13]. Pairing it with a detector-free comparator turns the gap into two additive terms, which tells a practitioner which component to examine: a box-prompted pipeline that degrades on new data in the way BraTS-Africa does calls for examining the detector before replacing the segmenter; on RHUH-GBM, the smaller increase was split between the two terms and resolved in neither. Re-calibrating the threshold is not enough. On the cohort where it helps, the best threshold, chosen on that cohort’s own test data, recovers about a quarter of the term, and on the other two cohorts, lowering it makes the term larger (Section 3.4).

**Why BraTS-Africa and not RHUH-GBM?** The two external cohorts differ from the held-out set in different ways (Table 1). BraTS-Africa was acquired at 1.5 T with 3–5 mm slices that were resampled to 1 mm by the distributors. Its FLAIR contrast index is lower than the other two cohorts, its T1ce contrast index is higher, and its tumors are larger and span more slices. RHUH-GBM was also acquired at 1.5 T, with FLAIR slices of up to 4 mm on its main scanner, so neither field strength nor slice thickness alone separates the two. The collapses that carry much of the BraTS-Africa term were associated with lower FLAIR contrast, lower tumor position, and, in some patients, an incomplete field of view (Section 3.4), which makes input quality and lesion location candidates. Four of the nine collapses lie low and near the midline, a location that the rule finds in none of the detector’s 258 training patients (Section 3.4); two of these four also have a truncated field of view, which can make a tumor appear low, so the two candidates are not separated.

The reference segmentations also differ, and this affects the two box arms unequally. One sign of this is that the BraTS2020 references have more connected components (median of 3.5 vs. 1.0; Table 1). The oracle-box arm takes its box from the reference it is scored against, so a more widely drawn reference costs it nothing. A detector trained to another cohort’s convention pays for the difference, and the whole cost falls in the detector-stage term. Compared with the held-out set, the BraTS-Africa reference has 1.63 times the median whole-tumor volume (Mann–Whitney *p* = 0.008) and 1.34 times the area per positive slice. For RHUH-GBM, where no increase was resolved, the ratios are 1.11 (*p* = 0.98) and 0.95. A volume ratio cannot distinguish a lesion that is larger from a reference drawn more widely around it, and delayed presentation has been reported in sub-Saharan Africa [23], so the lesions may genuinely be larger. Lesion size itself is unlikely to be the driver, because within each cohort, the term falls rather than rises with tumor volume (Section S11). With two external cohorts, none of these differences can be singled out as the cause, and separating annotation convention from acquisition would need both conventions applied to the same images.

**Sub-regions:** Detector-free models are credited with producing sub-regions that single-class box pipelines cannot [38,39]. For an enhancing tumor, the credit rests on a limit of single-box, class-agnostic prompting rather than on a measured advantage: a single tight box often cannot express the target, consistent with prompt-mode studies of the geometry a box imposes on the mask [5,37], so a box-prompted system that must deliver an enhancing tumor needs a different prompt rather than a better detector. For the tumor core, a correct box has clear headroom; what is missing is a trained detector, and the one trained here realized part of that headroom, with a resolved advantage only on RHUH-GBM, whose core label is narrower (Section 3.6).

**Seeds and leakage:** Seed sensitivity of the size found here has been reported for years [24,25,29,32]. On the tumor core in BraTS-Africa, detectors that differ only in training seed differ by as much as the two systems do, and the detector-free model varies across seeds as well (Section 3.6). On the whole tumor, the seed effect was far smaller than the gap. An end-to-end comparison on out-of-domain data should not rest on one training run; reporting a distribution rather than a point value is the established remedy [26,27,28]. The leakage mode described in Section 1 is easy to fall into: wherever the same public dataset is split independently for two studies, much of one test set is likely to sit in the other’s training set, and the literature shows that leakage is already a frequently reported flaw [33,34,35,36]. Here, the overlap was found when the two models were first compared, not anticipated (Section S5). The mutually held-out intersection is an inexpensive remedy.

### 4.1. Practical Implications

**When detector-stage failures matter:** Every three-cohort result here concerns the whole tumor: the union of the enhancing tumor, the necrotic and non-enhancing core, and the surrounding fluid-attenuated inversion recovery (FLAIR) hyperintensity. The whole tumor has clinical uses: tumor volumetry; T2/FLAIR-based target volumes in radiotherapy, for example, in lower-grade and isocitrate dehydrogenase (IDH)-mutant glioma, whereas current guidance for IDH-wild-type glioblastoma bases the target on enhancement and the resection cavity [49]; and the assessment of non-enhancing, mainly IDH-mutant, tumors under Response Assessment in Neuro-Oncology (RANO) 2.0 [50,51]. These cohorts are dominated by enhancing high-grade tumors, so the last use is scarcely represented here. Radiotherapy planning and response assessment use postoperative and follow-up imaging, while every scan here is preoperative, so these uses are named only to locate the whole tumor clinically, not as settings that were tested. On the whole tumor, the mean shifts are small compared with how precisely the reference is drawn. Human raters agree on the BraTS whole tumor at approximately 0.85 Dice [1], and every arm reaches at least this value in the domain. The detector-stage term is 0.006 to 0.091 Dice for the mean and 0.005 to 0.016 for the median patient (Section 3.2). What would change a decision is the failure mode, not the mean. The pipeline falls to 0.7554 on BraTS-Africa, 0.095 below the agreement level, because a few patients collapse; those collapses have no counterpart in the oracle-box arm, and the pipeline emits no confidence of its own for the segmentation. These would matter wherever the output is used without review, for example, in automated tumor volumetry. Partial omission matters there too: among the 86 BraTS-Africa patients who do not collapse, the pipeline’s whole-tumor volume is off by more than 20% in 21 patients, versus 2 for the detector-free arm (Section S17.9). No radiologist assessed the outputs or the input quality, and no reader study was done (Section 4.2). An agreement level and a paired difference are not the same quantity, so this comparison sets a scale, not a threshold.

**A coverage check as a hypothesis for prospective testing:** The check that uses only the detector’s output caught 8 of 9 collapses, with 9 false alarms in 86 cases on BraTS-Africa, so that fewer than half of its flags (8/17) were collapses. In addition, it raised no flag on the other two cohorts, which had no collapses (Section 3.4). Its form was chosen after the collapses had been seen, and only its cut-off came from the held-out set. It sees omission failures only, not boxes on the wrong structure, and a run length in slices penalizes small tumors by construction. It is cheap and could serve as a “send for review” trigger, but it is not a quality guarantee and needs prospective validation before use.

**Choosing between the two families:** The results suggest four criteria. They rest on one system of each kind and are not a comparison of architectures. (i) *Target region:* For an enhancing tumor, a single tight box is the wrong prompt. For the tumor core, a correct box has clear headroom over the detector-free model (Table 7), but a trained detector realized a resolved advantage only on RHUH-GBM, whose core label is narrower. For whole tumors, the detector-free model was more accurate here on every cohort. (ii) *Resources:* The staged design holds 491 MiB of weights versus 3221 MiB and takes 33.5 s per patient for a whole tumor versus 76.7 s on the same GPU. By our estimate, it would be the slower of the two if all three regions were needed (Section S3). (iii) *How each fails, and how that can be checked:* The pipeline fails by omission: it misses tumor-bearing slices, which a coverage check can flag. The detector-free model fails by addition: small components far from the lesion, which barely change Dice but raise HD95 above 20 mm in up to 39% of patients; keeping the largest connected component removes most of that tail, although it would also remove true tumors in multifocal disease (Section 3.1). Both kinds of error need review: missed slices leave tumors out of a volume, and detached islands create spurious targets. (iv) *Validation:* because the size of the detector-stage term depends on the cohort and on the training seed, whichever design is chosen should be measured on the target population before use, with human oversight in either case.

**Boundary error and enhancing-tumor volume:** The oracle-box arm’s median whole-tumor HD95 is 4.7 to 7.3 mm versus the pipeline’s 6.2 to 10.6 mm (Table 3). The BraTS-Africa pipeline figure is over the 92 patients on which it produces a surface at all, so it understates the gap, and the oracle-box arm is prompted only on positive slices, which favors it on a distance metric (Section S2). Under a perfect box, the oracle-box arm still over-estimates enhancing-tumor volume by a median 41% to 50% under the one-box protocol, and by 26% to 41% under one box per component (Section S7). RANO [50] assesses enhancing disease bidimensionally, and RANO 2.0 [51] also allows volumetric assessment, with a 40% increase in enhancing volume as its threshold for progression. An over-estimate of 41% to 50% is of that size, so a bias that differed between time points could change the response category; however, it was not measured here. For volumetric enhancing-tumor measurement, an over-estimate of that size rules this prompt protocol out, however good the detector becomes.

### 4.2. Limitations

- **On RHUH-GBM, every arm is run under a corrected brain mask rather than the detector-free model’s published preprocessing.** All arms were re-run the same way so that they see the same volumes (Section 3.2). That is the primary analysis, and it departs from what the model’s authors specify. The published preprocessing changes the gap on that cohort and the sign of the residual at adapter seed 42, and leaves the detector-stage term untouched (Table S10).
- **The comparison is not architecture-controlled, and the base model’s exposure is only partly documented.** The two systems differ in architecture, size, and pretraining corpus. The configuration shipped with the checkpoint [38] lists BraTS MRI under BraTS 2023 sequence names, so the held-out patients were likely included in pretraining, and BraTS-Africa may have been as well.. This affects the residual, the gap, and Table 7, not the detector-stage term; because it would raise the in-domain detector-free score, it would also push the share of the increase reported in Section 3.2 upwards. Table 6 measures the model difference at a fixed box, but that term carries pretraining, capacity, and the fitted adapter together, so no ordering of the two systems is drawn from it (Table 2, Section S9). The detector-stage term does not involve this arm.
- **The in-domain reference is small and was used in model selection, which is the main internal-validity limitation.** Neither the 24 shared patients nor the detector’s own 55-patient split is free of selection, and both biases favor the claim made here (Section 3.2, Sections S5 and S10). Against the cleanest baseline available, the BraTS-Africa contrast narrows but still excludes zero, while the RHUH-GBM contrast falls to +0.0026 and spans zero. The intervals are correspondingly wide: an in-domain detector-stage term of up to +0.0219 and an RHUH-GBM contrast of up to +0.0295 are compatible with the data. A clean reference would have required retraining the detector on a split drawn without reference to these patients.
- **Replication is uneven, and training is not bit-reproducible.** The detector-stage term is repeated at a second detector seed and the total gap at three adapter seeds (Section 3.5). Two seeds check the term rather than characterize it, and three points give a range, not a distribution [24,28], so the spreads in Section 3.6 are lower bounds. A same-configuration, same-seed repeat differs by 0.0005 F1 against a seed-to-seed variation of 0.0187 (Section S13), as reported for medical segmentation [29,32].
- **The prompt protocol and the threshold:** Under one box per connected component, every detector-stage term rises, the BraTS-Africa contrast is almost unchanged, and the in-domain term becomes distinguishable from zero (Section S7). The categorical reading of the in-domain baseline is therefore protocol-dependent and is not relied on. The threshold was swept downwards from the deployed value on all three cohorts at five points. Thresholds above 0.55 were not tested, and the best value was chosen on test data and is reported as an exploratory bound (Section 3.4).
- **Sub-region labels differ in meaning across cohorts, and an enhancing tumor cannot be identified from the inputs, as the benchmark defines it.** RHUH-GBM places a non-enhancing tumor in its peritumoral label (Section S8). A whole tumor is unaffected, but every cross-cohort tumor-core or enhancing-tumor comparison carries a labeling-convention confound removable only by re-annotation. No enhancing-tumor detector was trained, so the argument that one would not help is an argument, not a measurement. BraTS defines an enhancing tumor relative to native T1, which no arm received, so none of them separated contrast uptake from intrinsic T1 hyperintensity. This applies equally to both box arms and leaves their contrast unaffected.
- **Boundary metrics are reported for the three main arms, with caveats.** The oracle-box arm is prompted only on positive slices, which favors it on a distance metric (Section S2). No arm received component filtering or other post-processing in the main analysis (Section S14), so the detector-free arm’s HD95 tail reflects its raw output, and NSD is computed on boundary voxels as an approximation (Section S2). Overlap alone would have missed that tail, which is the failure a distance metric is recommended to cover [52].
- **The cohorts are characterized only partly at the case level.** Scanner and sequence parameters are not available per case for any cohort, and BraTS-Africa releases no per-case clinical data. Table 1 therefore combines cohort-level statements from the source publications, per-case grade, age, and sex where released, and measurements on the released images. With seven low-grade patients in the held-out set, grade could not be examined as a cause. Tumor type is not matched: RHUH-GBM is a glioblastoma cohort (WHO 2016) that is 90% IDH wild-type; BraTS-Africa reports presumed glioma without pathological confirmation, so mimics such as metastasis, lymphoma, or infection cannot be excluded; and BraTS2020 mixes grades. The references also differ in kind. The BraTS2020 references are manual, or manually revised after semi-automatic pre-segmentation [2], and neuroradiologist-approved, while the two external references are algorithm-seeded and corrected by experts (Table 1). All scans are preoperative, and all contain a tumor: two RHUH-GBM patients are recurrent cases treated before their preoperative scan [22], and excluding them leaves the RHUH-GBM result unchanged (Section 3.2). No tumor-free or non-glioma scans were tested, so false-positive behavior on such scans is unknown. The detector processes slices independently; only a tight, unpadded oracle box with single-mask output was used; and the coverage check is defined on these cohorts. No radiologist reviewed the input images, the lesion locations, or the acceptability of the outputs.
- **Three cohorts do not establish a curve, and no dense-prediction baseline is reported.** The claim is categorical, not a dose–response relationship. A leaderboard figure is computed per case and per region on different splits, so quoting one would not be a like-for-like comparison. Running a dense model such as nnU-Net [53] is a study of its own, and each claim here is a contrast within one system, with the segmenter and patients held fixed.

## 5. Conclusions

A comparison’s magnitude should be read as a property of the cohort it was measured on until the protocol has been carried to external data, and of the training run it came from until that run has been repeated. Measured on two external cohorts and repeated across training seeds, the comparison of a box-prompted pipeline with a detector-free foundation model yields three results that a single end-to-end score would have hidden.

First, for whole tumors, the detector stage accounts for most of the increase in the gap on BraTS-Africa; on RHUH-GBM, a detector-stage cost is present, but no increase over the in-domain value could be resolved. The pipeline degrades on BraTS-Africa, while the oracle-box arm’s own movement is unresolved, which establishes that the pipeline degrades more, not that the oracle-box arm does not. This is a statement about these cohorts and this pair of systems, not a general law. It does not carry over to the tumor core, where the in-domain detector-stage term excludes zero at every detector seed, and RHUH-GBM falls below the held-out set (Section 3.6 and Section S16).

Second, the claim that detector-free models are the only route to tumor sub-regions is not supported for the tumor core, where a correct box has clear headroom; a trained detector realized a resolved advantage only on the cohort whose core label is narrower. For an enhancing tumor, a single tight box is a limit that a more accurate detector does not remove, although no advantage of either system on that region was established.

Third, on the tumor core, the difference between two training seeds of one system can be as large as the difference between the two systems. The in-domain/external contrast survives a change of detector seed. Two tumor-core findings are not stable across seeds: the size of the RHUH-GBM tumor-core gap varies across adapter seeds, and on BraTS-Africa, no detector seed resolves the question of which system is better. Such comparisons should be replicated before they are believed. For the same reason, the in-domain detector-stage term is not described as zero: two further checks find it to be above zero (Sections S7 and S16).

In practice, a box-prompted pipeline that loses accuracy on a new cohort should have its decomposition measured on that cohort before an architecture is chosen. Where it degrades, as on BraTS-Africa, the detector should be examined first; a check on the detector’s output coverage is a candidate safeguard to be validated prospectively.

## Figures and Tables

**Table 3:** Overlap and boundary agreement of the three arms.

Cohort	*n*	Oracle-Box	Pipeline	Detector-Free	Gap	Wins	Median HD95, mm (Oracle-Box/Pipeline/Detector-Free)	Mean NSD (Oracle-Box/Pipeline/Detector-Free)
BraTS2020 (shared held-out set)	24	0.8672	0.8612	0.9189	+0.0576	22/24	4.69/6.23/7.03	0.765/0.745/0.873
RHUH-GBM	39	0.8643	0.8463	0.9314	+0.0850	38/39	5.92/7.55/5.10	0.765/0.714/0.907
BraTS-Africa	95	0.8467	0.7554	0.8829	+0.1275	95/95	7.28/10.59/12.17	0.683/0.580/0.798

Volumetric Dice for whole tumor, pooled over voxels. Gap is detector-free minus pipeline, with 95% CIs [+0.0403, +0.0795], [+0.0705, +0.0987], and [+0.1061, +0.1522] and Wilcoxon *p* = 6.0 × 10^−7^, 6.1 × 10^−10^, and 2.6 × 10^−17^; wins count the patients on which the detector-free arm is ahead. The oracle-box arm is a reference, not a deployable system. HD95, 95th percentile Hausdorff distance; NSD, normalized surface Dice at 2 mm. On BraTS-Africa, the pipeline’s boundary figures are over the 92 patients for whom it produces a surface at all, which flatters the pipeline. Full boundary results are in Tables S2 and S16.

**Table 4:** Decomposition of the gap.

Cohort	*n*	Cohort Detection F1	Term	Value	95% CI	vs. Held-Out	95% CI
BraTS2020 (shared held-out set)	24	0.867	detector-stage	+0.0059	[−0.0078, +0.0219]	—	—
			residual	+0.0517	[+0.0368, +0.0671]	—	—
RHUH-GBM	39	0.909	detector-stage	+0.0180	[+0.0097, +0.0285]	+0.0120	[−0.0062, +0.0295]
			residual	+0.0671	[+0.0540, +0.0794]	+0.0154	[−0.0047, +0.0348]
BraTS-Africa	95	0.799	detector-stage	+0.0914	[+0.0542, +0.1338]	+0.0854	[+0.0450, +0.1303]
			residual	+0.0362	[+0.0029, +0.0645]	−0.0155	[−0.0515, +0.0166]

The gap between the pipeline and the detector-free comparator is split into a detector-stage term and a residual. The detector-stage term is the oracle box minus the pipeline, and the residual is the detector-free value minus the oracle-box value; the two add up to the gap in Table 3 by construction. “vs. held-out” is the difference from the same term in the domain. Intervals are 95% percentile bootstrap (20,000 draws, random number generator seed 1337) paired within a cohort; between cohorts, they are the difference of two independent means, with each cohort resampled separately (Section 2.6). An interval that excludes zero supports a difference in that direction; one that spans zero does not establish a difference and does not establish its absence. Detection F1 is the cohort figure at the deployed threshold of 0.55. Exploratory: BraTS-Africa minus RHUH-GBM on the detector-stage term is +0.0734 [+0.0351, +0.1162] at detector seed 1337, and +0.0591 [+0.0259, +0.0958] at seed 42.

**Table 5:** Primary contrasts of the detector-stage term.

Contrast	Mean Difference [95% CI]	Bonferroni 97.5% CI	Holm-Adjusted *p*	Difference of Medians [95% CI]	20% Trimmed Mean Difference [95% CI]	Cliff’s δ [95% CI]
BraTS-Africa − held-out	+0.0854 [+0.0450, +0.1303]	[+0.0396, +0.1370]	≤ 2.0 × 10^−4^	+0.0114 [+0.0031, +0.0251]	+0.0249 [+0.0089, +0.0478]	+0.36 [+0.15, +0.57]
RHUH-GBM − held-out	+0.0120 [−0.0062, +0.0295]	[−0.0094, +0.0320]	0.181	+0.0045 [−0.0027, +0.0178]	+0.0090 [−0.0011, +0.0192]	+0.26 [−0.03, +0.54]
BraTS-Africa − RHUH-GBM (exploratory)	+0.0734 [+0.0351, +0.1162]	—	—	—	—	+0.17 [−0.03, +0.36]

The external cohort minus held-out set under the multiplicity correction and the robust estimators; the last row is the exploratory contrast between the two external cohorts. Holm-adjusted *p* is the two-sided bootstrap *p* from the same 20,000 resamples as the mean-difference interval, adjusted over the two primary contrasts; ≤ 2.0 × 10^−4^ marks the smallest Holm-adjusted value 20,000 resamples can give. At detector seed 42, the two contrasts are +0.0695 [+0.0353, +0.1074] (Holm-adjusted *p* ≤ 2.0 × 10^−4^) and +0.0104 [−0.0052, +0.0258] (*p* = 0.191). The Mann–Whitney test on the per-patient terms agrees (Holm-adjusted *p* = 0.012 and 0.080). Median and trimmed-mean intervals are from Section S11. Cliff’s δ is the probability that a patient in the first cohort has the larger term minus the probability of the reverse.

**Table 6:** Prompt and model terms from the fourth arm.

Cohort	*n*	Fourth Arm	Term	Value	95% CI	vs. Held-Out	95% CI
BraTS2020 (shared held-out set)	24	0.9373	prompt	+0.0184	[+0.0120, +0.0263]	—	—
			model	+0.0701	[+0.0552, +0.0861]	—	—
RHUH-GBM	39	0.9512	prompt	+0.0198	[+0.0123, +0.0289]	+0.0014	[−0.0094, +0.0125]
			model	+0.0869	[+0.0754, +0.0991]	+0.0168	[−0.0028, +0.0363]
BraTS-Africa	95	0.9208	prompt	+0.0379	[+0.0246, +0.0552]	+0.0195	[+0.0040, +0.0387]
			model	+0.0741	[+0.0519, +0.0931]	+0.0040	[−0.0231, +0.0283]

The residual in Table 4 is split by giving the detector-free model the ground-truth box in addition to its region name. Prompt term: fourth arm − detector-free; model term: fourth arm − oracle-box; residual = model − prompt. Within a cohort, the interval is a percentile bootstrap on the mean paired difference; the last column is the unpaired contrast against the held-out set, computed as in Table 4 (Section 2.6).

**Table 7:** Tumor-core and enhancing-tumor headroom.

Cohort	Region	Oracle-Box (SAM 2.1-L)	Detector-Free	Headroom	Oracle-Box Volume Error
BraTS2020	TC	0.8913	0.7590	+0.1323	+1.3%
BraTS2020	ET	0.6699	0.6312	+0.0387	+46.3%
RHUH-GBM	TC	0.9074	0.7540	+0.1534	+1.6%
RHUH-GBM	ET	0.7176	0.7255	−0.0079	+49.7%
BraTS-Africa	TC	0.8520	0.6956	+0.1565	+2.8%
BraTS-Africa	ET	0.7095	0.6195	+0.0901	+41.0%

Oracle-box scores (SAM 2.1-L) on the tumor core and enhancing tumors against the detector-free arm. Headroom is the oracle-box score minus the detector-free arm at adapter seed 42, the seed used in every other table; it gives the largest tumor-core headroom on both BraTS cohorts. Across the three adapter seeds, the tumor-core headroom is +0.1285 (+0.1233 to +0.1323) in the domain, +0.1473 (+0.1312 to +0.1573) on RHUH-GBM, and +0.1454 (+0.1376 to +0.1565) on BraTS-Africa, with each seed’s paired 95% interval excluding zero; the enhancing-tumor headroom is +0.0060 (−0.0479 to +0.0387), −0.0467 (−0.1192 to −0.0079), and +0.0635 (+0.0119 to +0.0901). Positive values mean that the oracle-box arm, which also knows which slices contain the region, scores above the detector-free model. The volume error is the median over patients of the oracle-box mask error. BraTS2020 ET is over 22 patients rather than 24, since 2 have no enhancing tumor in the reference. RHUH-GBM uses a narrower core definition (Section S8), so its sub-region rows are not directly comparable with the other two cohorts.

## Data Availability

BraTS2020, RHUH-GBM, and BraTS-Africa are publicly available; the latter two through The Cancer Imaging Archive (RHUH-GBM: https://doi.org/10.7937/4545-c905 (accessed on 10 October 2026); and BraTS-Africa: https://doi.org/10.7937/v8h6-8x67 (accessed on 10 October 2026)). The analysis code, the per-patient result files, and the detector’s patient-split manifest are available at https://github.com/aykutkaskaya/box-prompted-glioma-decomposition (accessed on 10 October 2026) and archived at https://doi.org/10.5281/zenodo.22744090 (accessed on 10 October 2026), which resolves to the most recent version. Imaging, model checkpoints, and detector weights are not redistributed there; the repository records where each is obtained. The scripts and result files for all analyses, including those of Section S17, are included in the archive. Every number computed in this study comes from those files through those scripts, and none of it is transcribed by hand (Section S15). Split manifests, checkpoint hashes, the cohort verification, and the naming of the loss in the released artefacts are described in Section S15.

## Supplementary Materials

The following supporting information can be downloaded at: https://www.mdpi.com/article/10.3390/diagnostics16203391/s1. Sections S1–S17; Table S1: BraTS-Africa at lower detector confidence; Table S2: Boundary agreement of the pipeline and the oracle-box arm; Table S3: Inference cost per patient and model weights; Table S4: The detector-stage term split by where the detector failed; Table S5: The oracle-box arm under the two prompt protocols; Table S6: Where the growth of the detector-stage term comes from; Table S7: The detector-stage term under two segmenters; Table S8: The detector-stage term at two detector seeds; Table S9: The whole-tumor gap at three LoRA adapter seeds; Table S10: The detector-free arm on RHUH-GBM under two preprocessings; Table S11: Detector training configuration; Table S12: The LoRA adapter at three seeds; Table S13: Tumor-core detectors at three seeds; Table S14: Per-patient detector-stage term on the tumor core; Table S15: The confidence threshold on all three cohorts; Table S16: Boundary agreement of the detector-free arm; Table S17: RHUH-GBM before and after the preprocessing correction; Table S18: Cohort characteristics measured on the released images; Table S19: Effect sizes of every term; Table S20: The nine BraTS-Africa collapses; Figure S1: Detector-stage failures, case by case; Figure S2: Seed spread in both designs; Figure S3: The confidence-threshold sweep on all three cohorts. References [5,33,34,35,36,40,52] are cited in the Supplementary Materials.

## Abbreviations

The following abbreviations are used in this manuscript:

WT	whole tumor
TC	tumor core
ET	enhancing tumor
HD95	95th percentile Hausdorff distance
NSD	normalized surface Dice
IoU	intersection over union
LoRA	low-rank adaptation
MRI	magnetic resonance imaging
FLAIR	fluid-attenuated inversion recovery
BraTS	Brain Tumor Segmentation (benchmark)
SAM	Segment Anything Model
RT-DETR	real-time detection transformer
CI	confidence interval
RHUH-GBM	Río Hortega University Hospital Glioblastoma dataset
TCIA	The Cancer Imaging Archive
RANO	Response Assessment in Neuro-Oncology
IDH	isocitrate dehydrogenase
