# Supplementary material for: Detector-Stage Failures Account for Most of the Increase in the External Deficit of a Box-Prompted Glioma Segmentation Pipeline on BraTS-Africa: A Three-Arm Decomposition Across Two External Cohorts
Source: Diagnostics (Basel). 2026 Oct 18;16(20):3391. doi: 10.3390/diagnostics16203391 (PMC13703227; doi:10.3390/diagnostics16203391)
Supplement: Supplementary file 1 [file diagnostics-16-03391-s001.zip › diagnostics-4612433-supplementary.pdf]

Supplementary Materials

Supporting information for *Detector-stage failures account for most of the increase in the external deficit of a box-prompted glioma segmentation pipeline on BraTS-Africa: a three-arm decomposition across two external cohorts*.

Every value here is computed by the analysis scripts released with the paper, from the same per-patient result files. Abbreviations follow the manuscript: WT, whole tumour; TC, tumour core; ET, enhancing tumour; SAM, Segment Anything Model; RT-DETR, real-time detection transformer; LoRA, low-rank adaptation; IoU, intersection over union; HD95, 95th percentile Hausdorff distance; NSD, normalised surface Dice; FLAIR, fluid-attenuated inversion recovery.

S1. The detector confidence threshold on BraTS-Africa

The detector's confidence threshold was not re-tuned externally, and on BraTS-Africa its precision stays high while recall falls. Re-running the detector arm at lower thresholds gives the values in Table S1; Section S17.1 extends the sweep to all three cohorts.

Table S1. BraTS-Africa at lower detector confidence.

conf	pipeline	detector-stage	gap	collapses	P / R / F1
0.55	0.7554	+0.0914	+0.1275	9	0.898/0.720/0.799
0.45	0.7661	+0.0807	+0.1168	8	0.878/0.731/0.798
0.35	0.7753	+0.0714	+0.1076	5	0.837/0.751/0.792
0.25	0.7806	+0.0661	+0.1023	4	0.778/0.767/0.772
0.15	0.7787	+0.0680	+0.1042	4	0.673/0.777/0.722

Recall rises monotonically across the sweep while precision falls; the pipeline peaks and then declines, and F1 falls throughout.

The sweep was continued until it reversed, so the answer does not depend on where the sweep was stopped. Lowering the threshold helps down to 0.25 and then stops helping: at 0.15 the pipeline loses 0.0019 and the detector-stage term rises again, because the boxes that a threshold that low admits are increasingly wrong — precision falls from 0.898 to 0.673 while recall gains only 0.057.

Giving the detector its best available threshold recovers +0.0253, which is 28% of the detector-stage term and 20% of the total gap. That gain is forgone by not re-tuning externally, but it is not the whole term: at the best threshold the detector-stage term is still +0.0661, against +0.0059 in domain. The remainder is not a threshold artefact, and Section S17.8 shows that on the collapses the detector's scores on tumour-bearing slices are mostly far below the deployed threshold (median 0.18).

S2. Boundary agreement

Distances and voxel spacing. HD95 and the surface metric are computed on the voxel grid without reading the affine, which is correct here because every volume in all three cohorts is 240 × 240 × 155 at 1 mm isotropic spacing, verified before the metrics were run; on a volume with anisotropic spacing the code would report voxel counts as millimetres without detecting the error.

A boundary metric. Every value so far is overlap-based, and overlap cannot see a component sitting far from the lesion — the limitation the detector-free arm's own boundary results demonstrate (Section S17.2), and the reason Metrics Reloaded recommends pairing the two [52]. The two box arms were therefore re-scored with HD95 and a boundary-voxel approximation to normalised surface Dice at a 2 mm tolerance. The approximation departs from the standard definition: the boundary voxels of each mask are counted that lie within the tolerance of the other, unweighted, rather than weighting by surface area as the metric's original definition does, so oblique surfaces are over-represented and structures thinner than two voxels are entirely boundary. The tolerance is two voxels on a 1 mm grid, chosen for that reason rather than derived from inter-observer variability as Metrics Reloaded [52] recommends. No inter-observer boundary distance in millimetres has been published for these cohorts, so the tolerance is uncalibrated and the absolute values should not be read as a distance from human agreement; only the paired difference between the two arms is interpreted below. Both are computed on the reconstructed volume rather than per slice so that through-plane distance counts. BraTS volumes are 1 mm isotropic, so voxel units are millimetres. Patients on which an arm predicts nothing have no surface and no distance; they are counted, not scored. The result is given in Table S2. The detector-free arm, scored the same way, is reported in Section S17.2. The confidence-threshold sweep of Section S1 is extended to all three cohorts in Section S17.1.

Table S2. Boundary agreement of the pipeline and the oracle-box arm.

cohort	arm	n	HD95 median	HD95 mean	NSD median	no prediction	pipeline − oracle HD95	95% CI
BraTS2020 (shared held-out set)	oracle-box	24	4.69	7.30	0.7676	0	+2.63	[−2.89, +9.78]
	pipeline	24	6.23	9.93	0.7420	0		
RHUH-GBM	oracle-box	39	5.92	6.48	0.7691	0	+5.32	[+1.74, +9.93]
	pipeline	39	7.55	11.80	0.7132	0		
BraTS-Africa	oracle-box	95	7.28	8.17	0.6932	0	+5.87	[+3.83, +8.11]
	pipeline	92	10.59	14.24	0.6062	3		

All three cohorts, by 95th percentile Hausdorff distance and normalised surface Dice.

HD95 is the symmetric 95th percentile surface distance in millimetres; NSD is the unweighted boundary-voxel approximation to normalised surface Dice at 2 mm described above. Medians are given because surface distances are heavy-tailed; the interval is a percentile bootstrap on the mean paired difference over the patients where both arms have a surface.

The boundary metric agrees with the overlap result, but the agreement is weaker than it looks. The oracle-box arm is prompted only on slices the reference marks as positive, so it cannot place a component outside the lesion's extent — the failure a distance metric is most sensitive to. The comparison is structurally favourable to that arm, so what follows is consistency rather than corroboration. The paired HD95 difference between the two arms includes zero in domain and excludes it on both external cohorts, which is the same pattern Table 4 shows for Dice and the pattern the paper's claim rests on. NSD separates the cohorts the same way. Its paired difference between the two arms is +0.0194 [−0.0142, +0.0570] in domain, +0.0512 [+0.0319, +0.0727] on RHUH-GBM and +0.0943 [+0.0651, +0.1262] on BraTS-Africa — the in-domain interval spans zero and the two external ones do not, as with HD95. The paired difference is given rather than the gap between the two arms' medians — +0.0256, +0.0559 and +0.0870 — because that gap is not computed within patients. It is the larger figure on the two cohorts where every patient has a surface, and the smaller one on BraTS-Africa, where the paired difference is +0.0943 and the three patients without a pipeline surface leave the two medians on different sets. The two metrics disagree about nothing here, though they could have: a pipeline that placed a small mask far from the lesion would look similar to the oracle-box arm on Dice and much worse on HD95, and that is not what these cohorts show.

It also reproduces the finding that the oracle-box arm is not an upper bound. Measured by HD95, the pipeline beats it on 35 of the 155 patients where both arms produce a surface, against 41 of the same 155 by Dice. The two rates are close, but the patients behind them are not the same: 51 patients win on one metric or the other and 25 win on both. The agreement is therefore in how often a detector box is the better prompt, not in which patients — which is what two metrics computed from different properties of the same masks would be expected to show, and a reason to report both.

The three BraTS-Africa patients with no pipeline prediction are among the nine collapses of Section 3.4. They have no boundary and are excluded from the distances above, which flatters the pipeline: the arm is scored on the patients where it produced something.

S3. Inference cost

The cost per patient, measured on the same GPU within the same session, is given in Table S3:

Table S3. Inference cost per patient and model weights.

system	time	ms/slice	detector	segmenter	total weights
RT-DETR → SAM 1 ViT-B	22.8 s	169	63 MiB	358 MiB	421 MiB
RT-DETR → SAM 2.1-L	33.5 s	249	63 MiB	428 MiB	491 MiB
MedSAM3 + LoRA	76.7 s	568	—	3 215 MiB	3 221 MiB*

Rows are the two box arms and the detector-free arm.

Times are means over the patients each row was measured on, taken on the same GPU within the same session, and the weights are in MiB. The two box rows time the deployable pipeline — detector and segmenter over every usable slice, whole tumour only — and the detector-free row returns all three regions in the same pass, so the rows are not like-for-like; the ratios below are against the SAM 2.1-L row.

* The total includes the 6 MiB LoRA adapter, which has no column of its own because the detector-free arm has no detector.

What the columns are. The weights are model tensors, not files: the released MedSAM3 and LoRA checkpoints carry optimiser state, which a deployment does not hold, so their files are 9 555 and 18 MiB against the 3 215 and 6 MiB of weights, while the box arms' files carry weights only. Quoting file sizes throughout would have counted two different things and overstated the ratio threefold. Detector and segmenter are listed separately because the staged design needs both. The times come from the per-patient timings stored with the results rather than from a separate benchmark: 158 patients for the SAM 2.1-L and detector-free rows, 119 for the SAM 1 ViT-B row, whose RHUH-GBM timings predate the corrected normalisation and are not on the same footing as the rest of the column, so the ratio against SAM 1 below is taken on its own 119 patients, where the detector-free arm averages 73.8 s. The oracle-box arm is not timed here at all: it segments only the slices that carry tumour and is therefore faster than anything deployable.

The time advantage is a whole-tumour advantage and does not survive the sub-regions. On whole tumour the detector-free arm costs 2.29 times the wall-clock of the box pipeline, and against the lighter SAM 1 pairing 3.2 times. Those two are the only ratios in this section that the sub-region argument below does not overturn. That comparison is between one region and three. The detector-free arm returns all three regions in its 76.7 s; a box pipeline needs a further detector and a further segmenter pass per region. The second was measured: the tumour-core pipeline costs 30.6 s over the same 158 patients, so a two-region box deployment is 64.1 s and the ratio falls to 1.2. For the third region the oracle-box arm gives the increment directly — its own per-region means are 27.7 s for whole tumour, 47.0 s for two regions and 65.6 s for three, a factor of 2.37 — and applying that factor to the

pipeline row gives 79.4 s against the detector-free 76.7 s. At three regions the staged design is the slower of the two.

This is reported rather than the whole-tumour ratio alone because the sub-region question is one this paper raises itself. A deployment that only needs whole tumour is 2.29 times faster staged; a deployment that needs all three is slower. The time ratio also varies by cohort even at whole tumour, from 2.13 on RHUH-GBM through 2.26 on BraTS-Africa to 2.65 on the held-out set, so 2.29 is a pooled figure and not a property of any one cohort.

The memory advantage does survive, and it is the durable one: 491 MiB against 3 221 MiB, a factor of 6.6, independent of how many regions are requested, since the extra regions cost passes rather than weights. Against the SAM 1 pairing the factor is 7.7. Weight size is a crude proxy for deployability and is reported because it is what was measured; peak memory would be the more clinically meaningful figure and was not recorded.

S4. Threshold selection

The confidence threshold of 0.55 is inherited from the detector study and is not the optimum of either split: on the validation split the best threshold is 0.46 (F1 0.8626, against 0.8586 at 0.55) and on the test split it is 0.48 (0.8541). What the validation curve does establish is that choosing on test rather than on validation buys almost nothing — the validation-optimal threshold 0.46 scores 0.8537 on test against 0.8541 at the test optimum, an optimism of +0.0003 — and that the optimum is broad, the range 0.45–0.65 falling within approximately 0.005 F1 on validation. This sweep was run at stride 2 rather than the stride 1 used everywhere else in the paper, since it compares thresholds against each other on a fixed slice set rather than reporting a cohort figure; the F1 values above are therefore not comparable to the detection figures elsewhere, only to one another. The value 0.55 is kept because every other result in this paper was produced at it; what that choice costs is measured directly in Section S1, where sweeping the threshold downwards on the cohort where it hurts most recovers 28% of the detector-stage term.

S5. A leakage failure mode

Leakage between training and evaluation data is the most frequently reported methodological flaw in machine-learning-based science [33], and medical imaging supplies its own catalogue of instances [34–36]. The form encountered here is specific to comparing two independently trained models, and it was encountered rather than anticipated: both projects are the present authors' own, and the overlap came to light when the two models were first put on the same patients.

The natural comparison, which would be to run both systems on the 55 test patients of the detector study, is not valid. Both projects split the same 368 cases into 258/55/55 subsets but with different assignments, and **45 of those 55 patients are contained in the training set of the LoRA model.**

The usable comparison is the intersection of the two held-out sets: the patients that appear in neither model's task-specific training split, so that neither system is advantaged by that training (the base model's pretraining probably included them; Section 2.1). This is the held-out set throughout; the tables label it BraTS2020 (shared held-out set). The name should be read narrowly. It is held out of both models' *training*, which is what makes the comparison fair, but it is not held out of model selection: 14 of the 24 lie in the detector's validation split, over which its confidence threshold was chosen, and 13 lie in the adapter's. The other 10 fall in the detector's own 55-patient test split, which is what run 37 was selected on, so they are clean of the threshold choice but not of the run choice. No in-domain reference in this study is untouched by selection, and Section 3.2 estimates that bias rather than asserting that it is small.

Quantifying it works against the claim made in the paper, since the uncontaminated value is the *larger* denominator. The detector-stage term is −0.0008 on the 14 patients the threshold was chosen over and +0.0154 on the other 10, 2.6 times the pooled +0.0059. Those 10 are the cleanest in-domain reference available, being free of the threshold choice if not of the run choice. Measured against them the cross-cohort contrast narrows to +0.0026 [−0.0316, +0.0303] on RHUH-GBM and +0.0760 [+0.0260, +0.1270] on BraTS-Africa, and only BraTS-Africa still excludes zero. Ten patients is a thin basis, which is why the pooled 24 is the headline and this is the check on it. On this cohort (n=24) the detector-free arm leads by +0.0576 (95% CI [+0.0403, +0.0795], 22/24, $p = 6.0 \times 10^{-7}$). Measured with the leak present, on the detector's own 55-patient split, the gap reads +0.0909. The two figures differ by 0.033, but they are a rescoring over different patients as well as a change of leakage status — the two sets share only ten patients — so that difference bounds the leak rather than measuring it. It does not create the gap either way.

S6. What the detector-stage term is made of

The detector-stage term aggregates several things, as Section 2.5 says, and does not separate them. It can be separated. Define a hybrid arm that keeps the pipeline's own mask on every slice where the detector fired and substitutes the oracle-box mask where it did not. Because Dice is pooled over voxels, the identity closes exactly:

```
 (oracle_box − pipeline)  =  (oracle_box − hybrid)  +  (hybrid − pipeline)
detector stage              box geometry               missed detection
```

The second term is what a detector that never missed a slice would recover, which is what lowering the confidence threshold reaches for. The first is what is left: the mask the detector's own boxes produce where it did fire, against the mask a tight ground-truth box produces on the same slice. That first term is not a pure geometry effect — a box on the wrong structure lands there too — so it is an upper bound on what better placement and shape could recover rather than an estimate of it. The split also

depends on the order of substitution: missed slices are substituted first, and what is left is attributed to geometry. The split is given in Table S4.

Table S4. The detector-stage term split by where the detector failed.

cohort	detector stage	missed detection	box geometry	positive slices missed	tumour voxels on them
BraTS2020 (shared held-out set)	+0.0059	−0.0018	+0.0078	226 / 1 621	1.1%
RHUH-GBM	+0.0180	+0.0023	+0.0157	196 / 2 597	0.7%
BraTS-Africa	+0.0914	+0.0751	+0.0162	1 530 / 7 052	6.4%

Missed detection is what substituting the oracle-box mask on slices with no proposed box recovers; box geometry is what remains on slices where the detector did fire.

The two external cohorts do not split the same way, and the difference tracks the difference in their terms. On BraTS-Africa missed detection is 82.2% [68%, 93%] of the term: what grows out of domain is overwhelmingly slices on which the detector proposes nothing, not boxes placed poorly. On RHUH-GBM, where the term is small to begin with, missed detection is only 12.5% of it — the detector skips 196 of 2 597 tumour-bearing slices there, they carry 0.7% of the tumour voxels, and the oracle-box arm's own median Dice on them is 0.694, so they are slices a correct box would not have rescued either. What is left on RHUH-GBM is box geometry, +0.0157. Box geometry contributes +0.0157 and +0.0162 externally against +0.0078 in domain, but the between-cohort contrasts against it, unpaired as Section 2.6 requires, are +0.0080 [−0.0096, +0.0238] and +0.0085 [−0.0107, +0.0271], so box geometry cannot be distinguished from a constant across the cohorts, nor shown to be constant; what can be said is that it does not grow the way missed detection does. The slices the detector skips on BraTS-Africa are, by contrast, worth having: a ground-truth box scores a median 0.762 on them, close to what the oracle-box arm achieves over that cohort as a whole, which is why substituting it there recovers most of the term.

This also puts a number on the threshold experiment above. The best available threshold recovers +0.0253 on BraTS-Africa, which is a third of the +0.0751 that perfect recall would return. The remedy points in the right direction and reaches part of the way; the rest of the missed-detection term is out of reach of the threshold, because at 0.15 the boxes admitted are wrong often enough to cost more than they recover.

In domain the split reads differently, in a way that looks at first like a sign error: the missed term is slightly negative, substituting oracle-box masks on the slices the detector skipped making the pooled Dice marginally *worse*. Those 226 slices carry 1.1% of the tumour voxels, and the oracle-box arm's own median Dice on them is 0.594 against a cohort mean of 0.8672 — they are marginal cross-

sections at the ends of the lesion where a correct box does not help either, so inside the training distribution the whole of the detector's already negligible cost is box geometry.

Figure S1 illustrates what this failure looks like in practice. Ten BraTS-Africa patients are passed through both box arms on one axial slice each, with the same segmenter in both columns, so that only the source of the box differs. For BraTS_SSA_00129, 00180 and 00215 the detector proposes nothing at all and the volumetric Dice is exactly 0.000, whereas the oracle-box arm reaches 0.897, 0.909 and 0.858 on the same patients. Those three figures are volumetric, over the whole reconstructed lesion, and are not the per-slice values printed on the panels of Figure S1. Nine of the 95 patients fall below 0.5 through the pipeline, and none of them do so through the oracle-box arm. The remaining panels cover the rest of the range up to 00046, where the detector costs nothing at all: a volumetric 0.922 against an oracle-box 0.920. The segmenter performs adequately in all of these cases. Where no box clears the confidence threshold the panel carries no mask and is marked accordingly; a patient with a non-zero volumetric Dice may still have a slice on which no box was proposed.

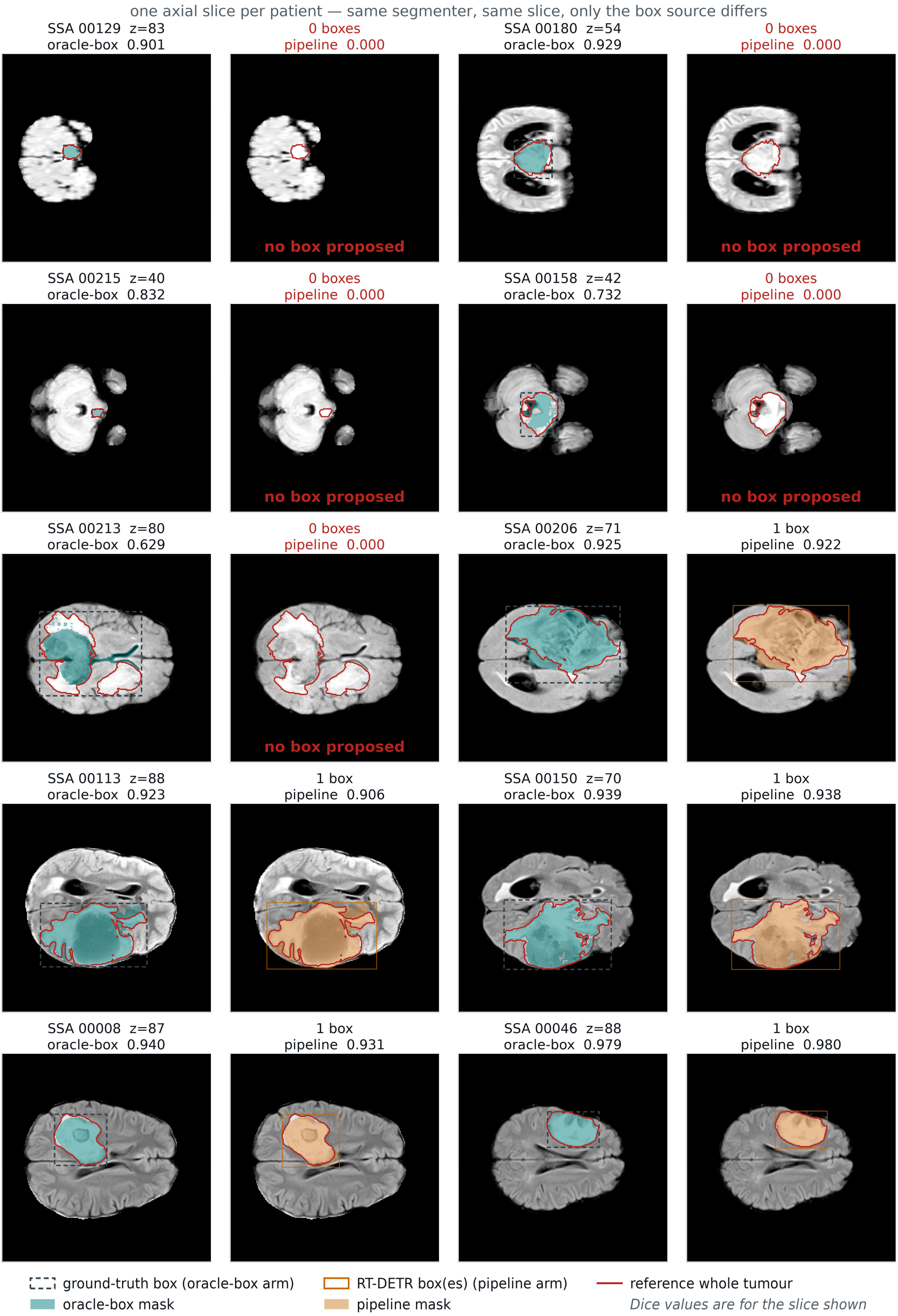

Figure S1. Detector-stage failures, case by case. Ten BraTS-Africa patients, one axial slice each, ordered by volumetric pipeline Dice; Figure 3 shows four of them. The panels follow the legend of Figure 3, and the Dice values are for the slice shown. Images: BraTS-Africa collection, The Cancer Imaging Archive (https://doi.org/10.7937/v8h6-8x67) [40], used under the CC BY 4.0 licence; the boxes, masks and outlines were added in this study.

Where the rest of this is measured. The threshold sweep is in Section S1 (Table S1), where the best available threshold recovers part of the term and the remainder is shown not to be a threshold artefact. The boundary reading of the same contrast is in Section S2 (Table S2), where the agreement is also weighed.

S7. The oracle-box arm's prompt protocol

The oracle-box arm was prompted with one tight box over the whole region on each slice; the pipeline arm passes every box its detector emits and unions the resulting masks. Where the target is a single connected component those are the same protocol, and where it is not they are not. Whole tumour is multi-component on 55.4%, 40.5% and 35.7% of its positive slices across the three cohorts, so this is not a corner case, and the asymmetry runs one way: the oracle-box arm cannot address a component separately and the pipeline arm can. Two readings depend on it — the size of the detector-stage term, which is the difference between those two arms, and whether the enhancing-tumour cap of Section 3.6 is a property of box prompting or only of a one-box oracle. The oracle-box arm was therefore re-run with one tight box per connected component of the reference, unioned exactly as the pipeline unions its own, and changed nothing else. Components below ten voxels are dropped, and a slice whose components are all below that floor falls back to the single whole-region box so that the comparison is never one box against none. That floor is permissive — a detector would not propose a box for a ten-voxel component either — so the per-component arm here is closer to the best a multi-box prompt could do than to what a detector could deliver. The comparison is given in Table S5.

Table S5. The oracle-box arm under the two prompt protocols.

cohort	region	one box	per component	difference	95% CI	prompts/slice
BraTS2020 (shared held-out set)	WT	0.8672	0.8923	+0.0251	[+0.0150, +0.0366]	1.48
	TC	0.8913	0.9098	+0.0185	[+0.0059, +0.0368]	1.11
	ET	0.6699	0.7471	+0.0772	[+0.0266, +0.1387]	1.39
RHUH-GBM	WT	0.8643	0.8833	+0.0190	[+0.0112, +0.0303]	1.35
	TC	0.9074	0.9290	+0.0217	[+0.0101, +0.0367]	1.17
	ET	0.7176	0.7408	+0.0232	[+0.0111, +0.0388]	1.28
BraTS-Africa	WT	0.8467	0.8699	+0.0232	[+0.0157, +0.0316]	1.33
	TC	0.8520	0.8881	+0.0361	[+0.0221, +0.0521]	1.24
	ET	0.7095	0.7508	+0.0412	[+0.0275, +0.0566]	1.43

SAM 2.1-L, same patients and same reference. Prompts per slice is the mean number of boxes the per-component protocol issues. Intervals are percentile bootstraps on the mean paired difference.

The per-component prompt raises the oracle-box arm on every region, and the consequences are not the same for the two readings.

For the **enhancing-tumour cap**, the effect is real and partial. Enhancing tumour gains +0.0772, +0.0232 and +0.0412 Dice and its median volume error falls from +46.3%, +49.7% and +41.0% to +32.0%, +40.9% and +26.3%. That is a material improvement, and it locates what Section S17.7 could not: that section reports that fragmentation does not explain the cap, and the per-component protocol nonetheless removes the part of the enhancing-tumour volume error that fragmentation contributes, leaving box coincidence as the operative mechanism. But the cap does not disappear: the enhancing arm still over-fills by +32.0%, +40.9% and +26.3% where whole tumour and tumour core, under the same protocol, sit at −2.0%, −0.7% and −0.9% and at −1.3%, −0.1% and −0.9% respectively. For the **detector-stage term** the consequence is more serious, and it works against the paper. On BraTS2020 (shared held-out set) the term moves from +0.0059 (Table 4) to +0.0310 [+0.0169, +0.0502]. On RHUH-GBM the term moves from +0.0180 (Table 4) to +0.0370 [+0.0225, +0.0567]. On BraTS-Africa the term moves from +0.0914 (Table 4) to +0.1145 [+0.0789, +0.1554]. The one-box oracle understates the cost of having to find the box, because it handicaps the arm that stands in for a perfect detector. This does not touch the pipeline arm and so changes no deployable figure, but it does bear on the in-domain baseline against which the external cohorts are contrasted, and it bears on it in the unhelpful direction: the cohort with the most fragmented whole tumour is the in-domain one, so the one-box protocol understates the in-domain term by more than the others.

The correction is close to uniform: it adds +0.0251, +0.0190 and +0.0232 to the oracle-box arm on the three cohorts respectively, so the quantity the paper rests on — the contrast of each external cohort against the held-out set — is left almost where it was. Against the held-out set BraTS-Africa moves from +0.0854 to +0.0835 [+0.0430, +0.1280], excluding zero under both protocols, and RHUH-GBM from +0.0120 to +0.0060 [−0.0182, +0.0300], spanning it under both; the one-box intervals are those of Table 4. The central result of this paper is therefore not an artefact of the prompt protocol, and neither is the finding that no increase is resolved on the second external cohort.

The in-domain term depends on the protocol. Under one box per component the in-domain term is +0.0310 with an interval excluding zero, so the detector does not cost nothing inside the training distribution; it costs about a quarter of what it costs on BraTS-Africa. The categorical form of the claim — that the term is indistinguishable from zero in domain and substantial outside it — belongs to the one-box protocol. The quantitative form, which is what the decomposition was built to support, holds under both.

Every other value in this paper is from the one-box protocol, which is the protocol the term was defined under and is stated wherever it matters. Both are reported rather than one replacing the other: the

protocol is a real degree of freedom, and the original choice of it was the conservative one for the detector-stage term and the permissive one for the enhancing-tumour claim.

S8. Cohort preparation and scoring rules

Label conventions and the remap. RHUH-GBM encodes 1/2/3 as necrosis, peritumoural signal alteration — which its descriptor defines as including both oedema and non-enhancing tumour — and enhancing tumour; BraTS-Africa follows the BraTS 2023 definitions, whose three classes are non-enhancing tumour core (NETC), surrounding non-enhancing FLAIR hyperintensity (SNFH) and enhancing tumour (ET); BraTS2020 uses 1/2/4 for NCR/NET, ED and ET. The files obtained carry 1/2/3 in both external releases, so the remap is 3 → 4 in both cases. Without it every ET score would read zero and tumour core would silently lose its enhancing component. The remap aligns the values but not, for one cohort, the semantics. Whole tumour is the union of every non-zero label under all three conventions and is therefore unaffected, which is the region the three-cohort result of Section 3.2 is measured on. Tumour core is not: taken as labels {1, 4}, it includes the non-enhancing tumour in BraTS2020 and in BraTS 2023, where label 1 is the entire non-enhancing core, but not in RHUH-GBM, where label 1 is necrosis alone and the non-enhancing tumour sits in label 2 with the oedema. Tumour core and enhancing tumour are thus defined on a slightly narrower target in RHUH-GBM than in the two BraTS cohorts, and the sub-region comparisons of Section 3.6 carry that difference of labelling convention as a confound (Section 4.2).

Excluded cases. Of the 369 cases in the BraTS2020 release, 368 were used: BraTS20_Training_355 carries no segmentation file under the naming convention of the distribution obtained here and was excluded at ingest, before the split was drawn. Of RHUH-GBM's 40 patients, RHUH-0028 was excluded because its preoperative study is off the BraTS grid, at 230 × 230 × 138 with a non-axis-aligned affine.

Geometry. Both external cohorts are already on the 240 × 240 × 155, 1 mm BraTS grid, so no resampling was applied; volumes that did not match were refused rather than resampled.

The background mask. Two of this study's own rules took skull-stripped background to be exactly zero. That is true of BraTS2020 and BraTS-Africa and false of RHUH-GBM, which ships z-score normalised with a negative constant background, so that *intensity* > 0 there thresholds at the volume mean. The first rule discarded a median 43%, 46% and 62% of the brain in the three input channels, and 62% of the whole-tumour voxels in the contrast-enhanced T1 (T1ce) channel. The second, the slice-admission rule of Section S14, cost usable slices on 33 of the 39 patients, a mean of 3.06 over those 33 and 2.59 over the cohort. Both rules were corrected to derive the background from the volume, and their output is bit-identical to the old rules' wherever the background is already zero, as verified on every slice tested in the other two cohorts.

Discarding half the image made the oracle-box arm's task easier and the detector's harder, so the correction did not fall evenly. Re-run on the 39 patients, the oracle-box arm moves from 0.8963 to 0.8643 and the pipeline from 0.8271 to 0.8463. The detector-stage term falls from +0.0692 to +0.0180 and its contrast against the held-out set from +0.0633 [+0.0191, +0.1221] to +0.0120 [−0.0062, +0.0295], which spans zero; a second detector seed gives +0.0164. The slice rule alone moved the term by 0.000026 on the uncorrected images and is not an independent result. The detector-free arm is fed through the adapter's own normalisation, mirrored verbatim here and carrying the same flaw, so it too was re-run rather than leaving the three arms seeing different volumes; at adapter seed 42 it rises from 0.8508 to 0.9314, +0.0806 [+0.0485, +0.1234] paired, on 38 of the 39 patients. The three readings the published preprocessing makes anomalous — a total gap spanning zero at every adapter seed, a residual negative at seed 42, and a 0.0279 adapter-seed spread — are consequences of that mask and not of the cohort (Table S10; Section S12).

Intensity. Intensity was not reconciled, and the reason is narrower than it may look. Both normalisations used here (per-slice scaling by the 1st and 99th non-zero percentiles for the box arms; a volume z-score clipped to ±3 for the detector-free and fourth arms, Section S14) cancel any monotone affine map applied to the whole volume: a release that differs from this one by a scale and an offset arrives at the segmenter identically. That covers the difference in stated normalisation between these releases. It does not cover a non-affine difference — bias-field correction, histogram matching, or a different skull-stripping mask would change the percentiles themselves and would not cancel. No test for such differences was run. Because the two normalisations differ, such an effect could reach the residual and the model term; the detector-stage term compares two arms with identical preprocessing and is unaffected.

Empty regions. Patients whose ground truth contains no tissue in a region are excluded from that region's mean and reported separately, because the two arms do not treat an absent region alike: the oracle-box arm is scored only over the slices where the region is present, so a patient with no enhancing tissue gives it an empty prediction against an empty reference and a Dice of 1.0, while the detector-free arm is scored over every usable slice and receives 0.0 for the same patient. Neither value is meaningful, and the filter removes both — but a mean computed without it would flatter the reference arm and penalise the deployable one. Two of the 24 held-out patients have no enhancing tissue, and none of the external patients do.

Bootstrap seeds. The generator seed is fixed in each analysis script and recorded there rather than chosen per run; it is not the same seed in every script, so two scripts computing the same quantity would differ in the fourth decimal. Where a quantity appears in more than one table a single computation is quoted wherever the tables were built together; where they were not, two tables may differ in the fourth decimal for this reason and for no other. Ratios and differences are computed from un-

rounded values and rounded once, so a figure recomputed from the rounded entries of a table may differ in the last digit. No conclusion in this paper turns on a fourth decimal.

S9. What the fourth arm's model term does and does not separate

The fourth arm of Section 3.3 measures a model term at a fixed box; it receives the region name as well as the box. Four qualifications bound what that term is, and none of them is removed by measuring it on three cohorts rather than one.

The model term is not architecture. MedSAM3 differs from SAM 2.1-L in medical pretraining, in capacity — 7.5 times the weights — and in carrying a LoRA adapter fitted on BraTS2020, and this experiment separates none of those from each other. No ordering of the two models is drawn from it (Section 4.2).

Neither term is a main effect. The design has three of the four cells a prompt-type × model table would need, since SAM 2.1-L takes no text prompt: the prompt term (what adding a box gains) is measured within MedSAM3 only, and the model term at the box prompt only, MedSAM3 also receiving the region name there, so an interaction between them would be invisible here.

The adapter's exposure inflates the model term. Thirteen of the 24 held-out patients sit in the adapter's own validation split, which raises the MedSAM3 side by an unknown amount in the direction that makes the term larger — the caveat Section 3.2 records for the total gap, applying here with more force, this being the contrast that exposure would move most. The two external cohorts are not affected, neither being part of any training or selection here.

All three cohorts are measured at one adapter seed. The replication of Section 3.5 covers the deployable arms, not this one.

S10. The selection of detector run 37 and what it is worth

The detector's own 55-patient test split was not trained on, but it was used for selection: run 37 is the highest test F1 of the 37 ablation runs (0.8185, against a mean of 0.7792 and a standard deviation of 0.0454), measured on exactly these patients. The in-domain reference of Section 3.2 is therefore compromised by that choice as well as by the threshold choice, and the question is what the run choice is worth.

That optimism is real in detection F1 but does not transfer to the volumetric scale, where the oracle-box arm cancels and the pipeline arm carries the whole between-run difference: paired over the identical 55-patient set, run 37 stands +0.0009 above the mean of the other 36, with a bootstrap interval of [−0.0040, +0.0047] spanning zero, and ranks 17th of 37 on volumetric Dice. A signed-rank test on those differences returns $p = 0.009$ against $p = 0.70$ on the mean, and the mean is used throughout;

these per-run figures follow the ablation's own protocol and are not interchangeable with Table 4 in absolute terms.

Scored over the whole 55 rather than the 24 the decomposition uses, the oracle-box arm reaches 0.8366 and the pipeline 0.8328, a detector-stage term of +0.0038, 95% CI [−0.0060, +0.0142] — smaller still, but a rescoring over different patients rather than a replication, which is why Table 4 keeps the 24-patient figure.

The run choice therefore leaves the volumetric reference close to where any of the 37 would have put it, which is why Section S5 quantifies the threshold choice rather than the run choice. Neither is argued away: both are recorded in Section 3.2 as what makes the in-domain arm a selection-contaminated reference, and in Section 4.2 as the study's main internal-validity limit.

S11. What the detector-stage term survives: estimator, tail and lesion size

The interval, corrected for skew. A percentile bootstrap corrects neither median bias nor acceleration, and the per-patient term is right-skewed, so the same per-patient terms are also resampled independently and reported as bias-corrected and accelerated intervals: [−0.0061, +0.0253] in domain against the percentile [−0.0078, +0.0219], [+0.0109, +0.0321] on RHUH-GBM against [+0.0097, +0.0285], and [+0.0585, +0.1412] on BraTS-Africa against [+0.0542, +0.1338]. Every interval shifts slightly upwards and none changes what is read from it: the in-domain term still spans zero and both external terms still exclude it. The percentile intervals are quoted in the manuscript because they are the ones every other figure in the paper uses.

The contribution of the tail. The per-patient term is heavily skewed — on BraTS-Africa the top five of 95 patients carry 47% of it — so the cohort mean is not a typical patient's experience. Against the held-out set the BraTS-Africa contrast is +0.0114 [+0.0031, +0.0251] on the median and +0.0249 [+0.0089, +0.0478] on a 20% trimmed mean, both excluding zero as the mean +0.0854 does; on RHUH-GBM it is +0.0045 [−0.0027, +0.0178] and +0.0090 [−0.0011, +0.0192] against a mean of +0.0120, and no estimator separates it from zero. Dropping the patients on which the pipeline collapses below 0.5 Dice changes only BraTS-Africa, the other two cohorts having none: the term there falls to +0.0406, against an unchanged +0.0180 and +0.0059, so the result is not a handful of collapses. Direction and separation survive every estimator; size does not — at the median the cost of having to find the box is 0.010 to 0.016 Dice against the 0.018 to 0.091 of the means, though the ordering of the two external cohorts, an exploratory comparison, is the same under every estimator. In domain the interval spans zero but admits a cost of up to +0.0219, more than the RHUH-GBM term itself. What separates the cohort types is the contrast interval, which excludes zero on BraTS-Africa and not on RHUH-GBM.

The oracle-box arm is a reference, not an upper bound. On the 41 of 158 patients where the pipeline scores *above* it (Section 3.2) it does so by as much as +0.138 Dice, consistent with the reported sensitivity of promptable segmenters to box geometry [5] and with the enhancing-tumour result of Section 3.6. It is nonetheless ahead of the pipeline in every cohort mean, so the decomposition is unaffected; but it is not ordered like the two deployable arms, and either arm can move the term, as Table S6 shows.

Table S6. Where the growth of the detector-stage term comes from.

cohort	change in term	oracle-box arm shift	95% CI	pipeline arm shift	95% CI
RHUH-GBM	+0.0120	−0.0029	[−0.0330, +0.0282]	−0.0149	[−0.0535, +0.0274]
BraTS-Africa	+0.0854	−0.0205	[−0.0476, +0.0088]	−0.1059	[−0.1577, −0.0539]

The table shows, for each external cohort, whether the growth out of domain comes from the pipeline falling or from the oracle-box arm rising.

Each external cohort is compared with the held-out set; the change in the term is the oracle-box arm's shift minus the pipeline's. Intervals are percentile bootstraps on a difference of two independent means, since the cohorts contain different patients.

The two cohorts do not tell the same story. On BraTS-Africa the term grows because the pipeline degrades: its arm falls by 0.1059 with an interval excluding zero, while the oracle-box arm moves by −0.0205 with an interval that does not exclude zero — so what is established is that the pipeline degrades more, not that the oracle-box arm does not degrade. On RHUH-GBM neither arm moves detectably and the term grows by only +0.0120, every interval in the row spanning zero, so no contribution of that cohort to the external contrast is resolved; its within-cohort term remains positive at +0.0180 [+0.0097, +0.0285], the detector costing something there but not more than in domain. Whatever makes BraTS-Africa difficult for the pipeline, it is not that its tumours are harder to outline once a correct box has been supplied.

Lesion size does not explain the cohort difference in the term. The readiest rival explanation for the detector-stage term is lesion size: a detector missing small tumours would produce a large term wherever tumours are small, making the cohort label incidental. The data do not support it. Within each cohort the term is *negatively* associated with tumour volume where it is associated at all (Spearman −0.431 on the held-out set, $p = 0.035$; −0.033 on RHUH-GBM, $p = 0.841$; −0.429 on BraTS-Africa, $p = 1.4 \times 10^{-5}$), while the external cohorts have *larger* tumours, by a median factor of 1.11 on RHUH-GBM (Mann–Whitney $p = 0.98$) and 1.63 on BraTS-Africa ($p = 0.008$); either sign alone would have to reverse to manufacture the result. The within-cohort figures are given rather than the pooled one (−0.294, $p = 2 \times 10^{-4}$), which mixes within- with between-cohort variation where the rival explanation

concerns the latter. At 24 and 39 patients this is a failure to reject, not a demonstration of equivalence, and it argues against the first covariate reached for rather than all those separating the three cohorts.

S12. Robustness: a second segmenter and the seed replicates

A second segmenter. The detector-stage term is a difference between two arms that share a segmenter, leaving open whether it is a property of SAM 2.1-L. Every patient was therefore also scored with SAM 1 ViT-B, a segmenter of the same family that differs in architecture, size and training data, and Table S7 gives the term under both:

Table S7. The detector-stage term under two segmenters.

segmenter	held-out set	RHUH-GBM	BraTS-Africa	RHUH contrast	Africa contrast
SAM 2.1-L	+0.0059 [−0.0078, +0.0219]	+0.0180 [+0.0097, +0.0285]	+0.0914 [+0.0542, +0.1338]	+0.0120 [−0.0062, +0.0295]	+0.0854 [+0.0450, +0.1303]
SAM 1 ViT-B	+0.0097 [−0.0040, +0.0267]	+0.0150 [+0.0077, +0.0236]	+0.0921 [+0.0549, +0.1339]	+0.0052 [−0.0132, +0.0213]	+0.0824 [+0.0421, +0.1273]

Both arms are re-scored end to end with each; the detector, the threshold and the patients are unchanged. Intervals are percentile bootstraps, paired within a cohort and unpaired for the contrast.

The in-domain interval spans zero under both segmenters, both external within-cohort intervals exclude it, and the BraTS-Africa contrast excludes zero while the RHUH-GBM contrast spans it. The two differ in architecture, size and training data, so the term belongs to having to find the box and not to the model that consumes it. Figure S2 shows the spread the seeds of each design produce, against the cohort ordering that neither of them follows.

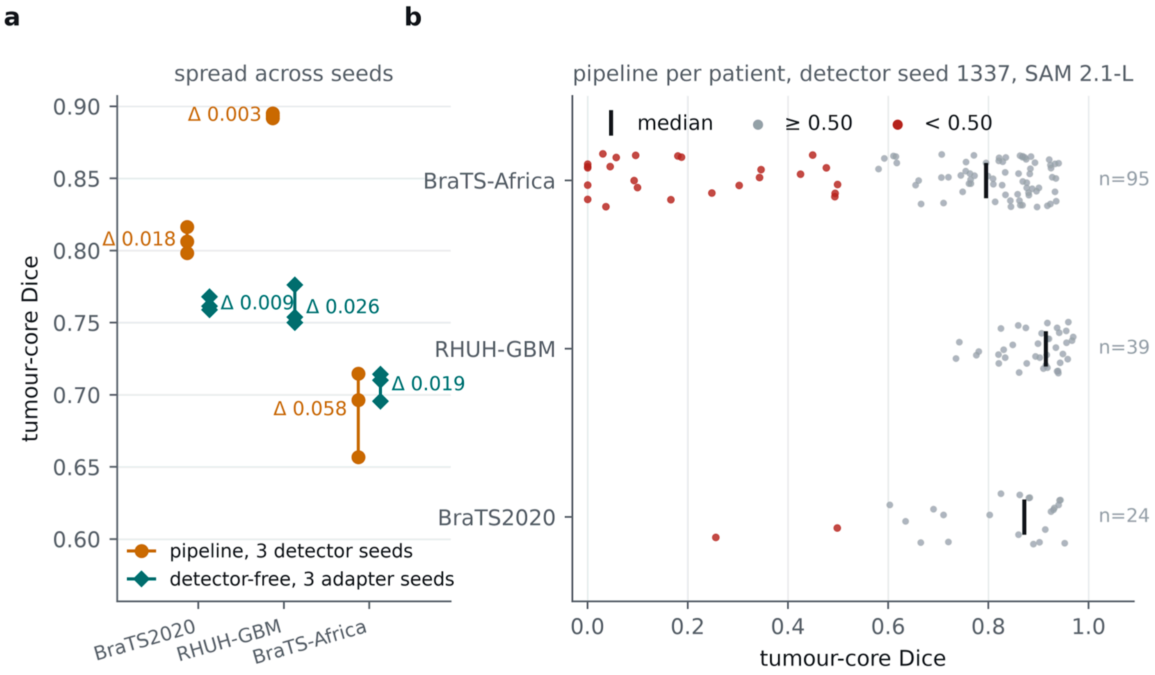

Figure S2. Seed spread in both designs. (a) Tumour-core Dice of the pipeline at three detector seeds (amber) and of the detector-free arm at three adapter seeds (teal): spreads of 0.018, 0.003 and 0.058 against 0.009, 0.026 and 0.019 on the held-out set, RHUH-GBM and BraTS-Africa, so neither design is reliably the noisier one (Section 3.6). (b) Per-patient tumour-core Dice of the pipeline at detector seed 1337, red below 0.5; the cohort mean is set by a tail of patients near zero.

A second detector seed. Out-of-domain comparisons should not be believed from a single training run, which puts this paper's own headline under the same obligation. The detector was retrained at a second seed and the pipeline arm re-run on all three cohorts; the oracle-box arm, prompted with the reference box, is unaffected, so both seeds are read against the same reference, patients being intersected across the runs (Table S8).

Table S8. The detector-stage term at two detector seeds.

cohort	n	seed 1337	seed 42	95% CI, seed 42
BraTS2020 (shared held-out set)	24	+0.0059	+0.0060	[−0.0057, +0.0197]
RHUH-GBM	39	+0.0180	+0.0164	[+0.0087, +0.0262]
BraTS-Africa	95	+0.0914	+0.0755	[+0.0440, +0.1123]

The intervals of Table 4 are recomputed here for the second seed.

The two detectors differ by 0.0069 in run detection F1 (threshold 0.25, the detector's own test split), not in the cohort figure of Table 4.

Both the contrast and the ranking survive the change of seed. The two seeds give +0.0059 and +0.0060 on the held-out set — closer than the width of either interval, so agreement in sign and not a precision claim — with the interval still spanning zero; on BraTS-Africa the term exceeds it by 0.07 Dice, its interval and its contrast against the held-out set (+0.0695 [+0.0353, +0.1074]) both excluding zero; on

RHUH-GBM the term is +0.0164 and the contrast +0.0104 [−0.0052, +0.0258], spanning zero. In this exploratory comparison BraTS-Africa costs more than RHUH-GBM at seed 1337 (+0.0914 against +0.0180) and still does at seed 42 (+0.0755 against +0.0164), a ranking that did not survive before the RHUH-GBM correction (Section S17.3). The detector therefore costs substantially more outside the training distribution than inside it at both seeds — though the in-domain term is not shown to be zero either (Section S7, one box per component) — while no reading of the three cohorts as a gradient survives at all.

Three adapter seeds. The same obligation applies to the other deployable arm. The detector-free model was run at three LoRA adapter seeds on all three cohorts under the frozen protocol; the pipeline and oracle-box arms do not depend on the adapter, so all are read against the same reference, patients intersected across the runs (Table S9).

Table S9. The whole-tumour gap at three LoRA adapter seeds.

cohort	n	seed 42	seed 52	seed 62	spread
BraTS2020 (shared held-out set)	24	+0.0576 [+0.0403, +0.0795]	+0.0608 [+0.0447, +0.0806]	+0.0614 [+0.0432, +0.0840]	0.0038
RHUH-GBM	39	+0.0850 [+0.0705, +0.0987]	+0.0911 [+0.0776, +0.1052]	+0.0889 [+0.0731, +0.1043]	0.0061
BraTS-Africa	95	+0.1275 [+0.1061, +0.1522]	+0.1265 [+0.1078, +0.1473]	+0.1258 [+0.1055, +0.1487]	0.0017

Detector-free minus pipeline, with 95% percentile bootstrap intervals on the paired difference. The pipeline and oracle-box arms are unchanged across the three.

The three seeds agree to 0.0038, 0.0061 and 0.0017 and every interval excludes zero, so on no cohort is the gap an artefact of one adapter. The instability RHUH-GBM showed under the model's published preprocessing, a spread of 0.028 against a point estimate of +0.0045, was a consequence of the brain mask and does not survive the correction (Section 3.2). No ratio between the two external cohorts is reported, the in-domain denominator it would divide by not being distinguishable from zero. The residual is positive on every cohort at every seed, on RHUH-GBM at +0.0671, +0.0731 and +0.0709, so the reading in Table 4 — that no change in the residual is resolved — does not depend on which adapter was used; the detector-stage term does not involve this arm at all. Table S10 gives that arm under both normalisations at all three adapter seeds, the corrected mask being the primary analysis of Section 3.2 and the model's published preprocessing the sensitivity.

Table S10. The detector-free arm on RHUH-GBM under two preprocessings.

adapter seed	Dice, published	Dice, corrected	gap, published	gap, corrected	residual, published	residual, corrected
42	0.8508	0.9314	+0.0045	+0.0850	−0.0135	+0.0671
52	0.8787	0.9374	+0.0324	+0.0911	+0.0144	+0.0731
62	0.8696	0.9353	+0.0233	+0.0889	+0.0053	+0.0709

Rows are adapter seeds; columns give the detector-free arm, the gap and the residual under the model's published preprocessing and under the corrected background mask.

The same 39 patients and the same box arms throughout. The corrected mask is the primary analysis of this paper and the published preprocessing the sensitivity; under the latter every gap spans zero — +0.0045 [−0.0383, +0.0388], +0.0324 [−0.0088, +0.0617] and +0.0233 [−0.0107, +0.0503] at the three seeds — and the residual at adapter seed 42 turns negative, −0.0135 [−0.0618, +0.0240]. The detector-stage term does not involve this arm and is unchanged at +0.0180 either way.

S13. Training configurations: the detector and the adapter

The configuration the detector was trained with is given in Table S11.

Table S11. Detector training configuration.

setting	value
architecture	RT-DETR-L (Ultralytics)
input size	640 × 640
optimiser	AdamW
initial learning rate	0.0005
weight decay	0.0001
epochs	100
early-stopping patience	50
batch size	12
mixed precision	yes
augmentation	fliplr 0.5, degrees 5, translate 0.05, scale 0.1
box loss	L1 + L_{ov} at α=0.50, weight 3.0
classification loss	focal
matching	Hungarian, IoU ≥ 0.5 for TP/FP
training seed	1337

Read from the resolved configuration the trainer wrote for run 37. The tumour-core detectors of Section 3.6 use the same configuration on tumour-core boxes, at seeds 1337, 42 and 62.

The detector-free arm is MedSAM3 with a LoRA adapter. The base checkpoint is used as released; the adapter was trained for this line of work, and its three replicates are seeds 42, 52 and 62. Inference for both arms, and the timings of Table S3, ran on a single NVIDIA GeForce RTX 5060 Laptop GPU in one session, in mixed precision, with the weights already resident so that no load time is counted. Table S12 gives the adapter's configuration, read from those checkpoints.

Table S12. The LoRA adapter at three seeds.

setting	value
base model	MedSAM3, used as released, frozen
adapted modules	attn.qkv and attn.proj in each of the 32 vision-backbone blocks
adapted layers / tensors	64 / 128
rank	8
trainable parameters	1 572 864
optimiser	AdamW, betas 0.9 and 0.999, decoupled weight decay
learning rate / weight decay	1×10^{-4} / 1×10^{-4}
epochs / optimiser steps	3 / 25 788
seeds	42 (primary), 52, 62
recorded digests	adapter state, patient split, configuration, dataset

Trained for the detector-free arm, at three replicate seeds. Read from the trained checkpoints.

The checkpoints themselves are not redistributed. The three replicates differ only in their seed, every other field being identical across them. Rank is the inner dimension of each adapter pair, and the parameter count is the adapter alone, the frozen MedSAM3 backbone excluded. The checkpoints do not record the adapter's scaling factor, dropout, batch size, learning-rate schedule or the prompt type it was fitted under, so those are not given; the last of them bears on the fourth arm of Table 6, which prompts that model with a box.

The detector used throughout. Every arm of this paper uses the same detector: RT-DETR run 37, trained with the overlap term of Section 2.2. Replication puts run-to-run variation at 0.0005 run detection F1 for a fixed seed and 0.0187 between seeds, which is the scale any claim about a design choice has to clear. The detector's configuration is in Table S11 and its cohort detection F1 in Table 4; Section S10 measures what choosing this run out of 37 is worth. Run 37 was selected in an ablation over 36 configurations in 37 runs, one configuration having been run twice; six of those configurations were re-trained at further seeds outside the ablation, which is where the seed-to-seed figure above comes from. The per-run summaries are released with the analysis code.

S14. Inference protocol

Figure 1 gives the shape of the two systems. This is what each stage receives and returns, at the level a reimplementation needs.

Input to the detector. One axial slice at a time, at native 240 × 240 resolution with no resizing or interpolation. Three modalities are stacked as the three channels of an 8-bit image, in the order FLAIR, T1ce, T2. Each channel is scaled independently by the 1st and 99th percentiles of its own non-zero voxels within that slice, clipped to that range and mapped to 0–255; voxels outside the brain stay at

zero. This reproduces the preprocessing the detector was trained on. RT-DETR is given the array in BGR channel order, since the Ultralytics wrapper reverses the channels internally.

Which slices. Every slice whose non-zero footprint across the three modalities reaches 500 voxels is evaluated; the rest carry no brain and are skipped. Stride 1 means every such slice, with no subsampling. The oracle-box arm produces nothing where its region is absent, so scoring it over the region's positive slices and over the whole evaluated set gives the same value.

From boxes to a mask. Detections below the confidence threshold are discarded. Every surviving box is used: each is passed to the segmenter separately and the resulting masks are combined by union, so a slice with three boxes contributes the union of three masks. No box is selected over another and none is merged beforehand. Boxes are passed to the segmenter exactly as the detector emits them, with no padding, dilation or rounding, and the segmenter is asked for a single mask rather than a set. No morphological post-processing, hole filling or component filtering is applied to any arm.

Detection F1, and the two of them. Matching is per slice, between the boxes that clear the threshold and the tight box of the ground-truth region on that slice, greedily from the highest-confidence prediction down, with a match requiring IoU ≥ 0.5. Each prediction matches at most one reference and vice versa. Counts are accumulated over every evaluated slice of every patient and the F1 is computed once from those totals, so it is a slice-level figure pooled over the group rather than a mean of per-patient values. One consequence bears on how weakly detection F1 tracks pipeline Dice (Section S17.7). The reference on a slice is a single tight box over the whole region, so a detector that emits one box per component is scored as one true positive and several false positives, while the masks of all of those boxes enter the Dice union. The matching therefore penalises exactly the behaviour that raises the segmentation score on a multi-component slice, and F1 is a correspondingly weak surrogate for what the pipeline arm is measured on.

Two figures in this paper carry that name and they are not interchangeable. **Cohort detection F1** is measured at the deployed threshold of 0.55, on the slices of the cohort being evaluated, and is what Table 4 reports. **Run detection F1** is what the training pipeline logs for a run: threshold 0.25, over every evaluated slice of the detector's 55-patient test split — 5 622 images for whole tumour and 4 046 for tumour core, of which 3 768 and 2 695 carry the region (Section S17.7). It is the figure quoted for the whole-tumour and tumour-core detectors in the Supplementary Materials (Section S13) and Section 3.6 and for the two detectors of Table S8. The two differ in threshold and in slice set, so a number from one series should never be compared with a number from the other. Where the distinction matters below, the threshold is named.

The detector-free arm. MedSAM3 receives the volume rather than one slice, as a pseudo-RGB stack of the same three modalities z-scored over their non-zero voxels and clipped to ±3, which is the normalisation its adapter was trained with — not the 8-bit form the detector receives. It is given one con-

cept prompt per region and no box. The three strings are `Whole tumor`, `Tumor core` and `Enhancing tissue`, one vision encoding serving all three. Only the last is an entry in the model's training query vocabulary; the other two are free text and reach the model through its byte-pair encoder. The adapter's vocabulary was not modified.

Scoring. Every arm is scored by volumetric Dice pooled over voxels, as defined in Section 2.4, against the same reference masks derived from the same label remapping.

S15. Released artefacts and verification

The loss is implemented and logged under the name `icarb` in the released artefacts, and the run directories, the experiment registry and the configuration hashes all use that identifier. The per-case grade, age and sex of the main-text cohort table come from files not included in the local image download: the RHUH-GBM clinical table from TCIA and the BraTS2020 name_mapping.csv and survival_info.csv, the latter from public copies that agree with each other; their sources are listed in the released archive. In this paper the term is denoted by L_{ov}.

The patient-level splits are identified by the hashes `fae05e5d…` and `f4c2efe1…`, and each LoRA checkpoint records `lora_state_sha256`, `split_sha256` and `dataset_sha256`, embedded in the checkpoint itself. Neither split hash is a digest of the released file: both are taken over a canonical JSON dump, and the adapter's recipe excludes the `split_sha256` field from its own input. A released script recomputes both and reports whether they match. The detector's split manifest is released and the adapter's is identified by its hash alone, so the leakage count of Section S5 is recomputable only on the detector side: 45 of the detector's 55 test patients fall in the adapter's training set, 6 in its validation set and 4 in its test set. The two projects drew 258/55/55 from the same 368 cases independently and, independently, excluded the same case for the same reason. The RHUH-GBM package was verified against its 720-file MD5 manifest (720 of 720), by a released script that writes the result rather than by a manual step. The case excluded before the split is listed with its reason in `excluded_patients.csv` alongside the split, so the 368 cases can be reconstructed exactly. One released key needs care when recomputing: `et_box_returns_tc` in `oracle_regions_summary.json` selects the patients whose enhancing-tumour prediction equals their tumour-core prediction — 0 of 22 on the held-out set, 32 of the 39 on RHUH-GBM and 58 of the 95 on BraTS-Africa — and reports 0.9094 against tumour core and 0.7356 against enhancing tumour on the RHUH-GBM set. The figures in Section S17.7 are over a different and narrower set — the 31 patients whose two ground-truth boxes are equal on every slice — and are 0.909 and 0.730. The two criteria agree on the conclusion and disagree in the third decimal; Section S17.7 uses the box criterion because the claim is about what a box can express. A separate script re-derives the values the released result files support — which is most of Tables 1–7 and of the supplementary tables from the archive alone, and all of them where the adapter project is checked out beside it; the adapter-side figures of Sections S5 and S9 need that second

checkout — and checks them in both directions: that every recomputed value appears in the text, and that every decimal quoted in the abstract, introduction, discussion or limitations — the sections that state no measurement of their own — matches something computed in Methods or Results at its own precision. It exits non-zero if either fails. The first direction alone is not sufficient: a value superseded by a re-run survives a check of that kind, because the number is still somewhere in the document. A third check requires every decimal number in the manuscript and the Supplementary Materials to match a value in the result files or in the output of the analysis scripts at its printed precision. The detector training configuration is given in Table S11, and Section S14 records the inference protocol.

S16. Tumour core: the detectors and the decomposition

Section 3.6 reads these runs against the oracle-box reference of Table 7; Table S13 gives them in full.

Table S13. Tumour-core detectors at three seeds.

cohort	detector seed	pipeline	retained	total gap	95% CI
BraTS2020 (shared held-out set)	1337	0.7984	30%	−0.0393	[−0.1146, +0.0433]
	42	0.8063	36%	−0.0473	[−0.1080, +0.0147]
	62	0.8163	43%	−0.0573	[−0.1272, +0.0174]
RHUH-GBM	1337	0.8918	90%	−0.1378	[−0.1834, −0.0942]
	42	0.8935	91%	−0.1395	[−0.1837, −0.0966]
	62	0.8952	92%	−0.1412	[−0.1857, −0.0976]
BraTS-Africa	1337	0.6569	−25%	+0.0387	[−0.0033, +0.0812]
	42	0.6965	1%	−0.0009	[−0.0389, +0.0383]
	62	0.7147	12%	−0.0192	[−0.0557, +0.0188]

Each detector was run through the same pipeline; the table gives the share of the Table 7 headroom each recovers. Its intervals use 10 000 resamples at generator seed 0.

The segmenter is SAM 2.1-L throughout. Retained is the share of the headroom of Table 7 the pipeline recovers; the total gap is the detector-free arm minus the pipeline, so a negative gap means the pipeline is ahead. The oracle-box scores are fixed per cohort at 0.8913, 0.9074 and 0.8520, and the detector-free arm at adapter seed 42 at 0.7590, 0.7540 and 0.6956; across its own three adapter seeds that arm gives 0.7590, 0.7614 and 0.7680 in domain, 0.7540, 0.7501 and 0.7761 on RHUH-GBM and 0.6956, 0.7144 and 0.7100 on BraTS-Africa.

The three detectors differ by 0.0098 in run detection F1, which is seed noise under any reading, and by 0.0578 in cohort Dice on BraTS-Africa. On that cohort the sign of the total gap is not stable across them.

Closing the decomposition on this region, rather than reporting the headroom alone, gives the detector-stage term there, in Table S14.

Table S14. Per-patient detector-stage term on tumour core.

cohort	detector seed	n	detector-stage term	95% CI	vs. held-out
BraTS2020 (shared held-out set)	1337	24	+0.0930	[+0.0422, +0.1610]	—
	42	24	+0.0850	[+0.0429, +0.1348]	—
	62	24	+0.0750	[+0.0321, +0.1300]	—
RHUH-GBM	1337	39	+0.0156	[+0.0002, +0.0312]	−0.0774 [−0.1480, −0.0232]
	42	39	+0.0139	[+0.0001, +0.0288]	−0.0711 [−0.1229, −0.0263]
	62	39	+0.0122	[−0.0038, +0.0297]	−0.0628 [−0.1213, −0.0159]
BraTS-Africa	1337	95	+0.1951	[+0.1421, +0.2510]	+0.1022 [+0.0155, +0.1802]
	42	95	+0.1555	[+0.1087, +0.2059]	+0.0706 [+0.0029, +0.1364]
	62	95	+0.1373	[+0.0933, +0.1853]	+0.0623 [−0.0079, +0.1274]

Values are given at three detector seeds on each of the three cohorts.

The term is the oracle-box arm of Table 7 minus the pipeline of Table S13. Intervals are paired within a cohort and unpaired between, from 20 000 draws at generator seed 1337 (Section 2.6). Patients whose reference carries no tumour core are excluded from the region's mean (Section S8). The whole-tumour equivalents are in Table 4.

Two readings of the whole-tumour result do not carry to this region. The in-domain term excludes zero at all three detector seeds, so the term being indistinguishable from zero inside the training distribution is a whole-tumour finding and not a general one; Section S7 shows the same claim failing under a matched prompt protocol, and this is a second way it fails. And RHUH-GBM falls below the held-out set rather than above it, the contrast excluding zero in that direction at all three seeds, where on whole

tumour it sat above and spanned zero. BraTS-Africa remains the higher of the two external cohorts on both regions, a comparison that is exploratory.

What survives both regions is the exploratory ordering of the two external cohorts against each other, and the size of the term on BraTS-Africa — the latter more weakly here, its contrast against the held-out set spanning zero at detector seed 62. What does not is the categorical in-domain baseline and the reading of the three cohorts as a gradient by provenance, which Section S12 already declined to make.

S17. Further analyses and detailed results

This section collects further analyses and detailed material referred to from the main text. Every number in it is computed by the released scripts, and every rerun was checked against the primary result files before use.

S17.1 The confidence threshold on all three cohorts

The pipeline was re-run on every cohort with the detector's boxes cached once at its score floor and each threshold applied offline. On BraTS-Africa this reproduces the separate runs of Table S1 exactly, patient by patient, at all five thresholds, and at 0.55 it reproduces the per-patient Dice and detection counts behind Table 4 on every cohort; the intervals of Table S15 come from a separate bootstrap draw and can differ from Table 4 in the fourth decimal. Lowering the threshold helps only on BraTS-Africa. On the other two cohorts every lower threshold enlarges the detector-stage term, and in domain thresholds of 0.25 and below introduce a collapse that 0.55 does not have (Table S15, Figure S3). The best BraTS-Africa threshold, 0.25, was chosen on that cohort's own test data and is an exploratory upper bound; the deployed 0.55 remains the primary analysis.

Table S15. The confidence threshold on all three cohorts.

cohort	threshold	pipeline Dice	detector-stage term [95% CI]	collapses	precision	recall
BraTS2020 held-out	0.55	0.8612	+0.0059 [−0.0078, +0.0219]	0	0.933	0.811
BraTS2020 held-out	0.45	0.8611	+0.0061 [−0.0076, +0.0218]	0	0.921	0.817
BraTS2020 held-out	0.35	0.8591	+0.0081 [−0.0063, +0.0254]	0	0.885	0.823
BraTS2020 held-out	0.25	0.8529	+0.0143 [−0.0019, +0.0347]	1	0.818	0.828
BraTS2020 held-out	0.15	0.8434	+0.0238 [+0.0048, +0.0473]	1	0.698	0.831
RHUH-GBM	0.55	0.8463	+0.0180 [+0.0095, +0.0287]	0	0.939	0.881
RHUH-GBM	0.45	0.8448	+0.0195 [+0.0108, +0.0306]	0	0.922	0.886
RHUH-GBM	0.35	0.8435	+0.0208 [+0.0123, +0.0312]	0	0.900	0.895
RHUH-GBM	0.25	0.8414	+0.0229 [+0.0144, +0.0329]	0	0.856	0.901
RHUH-GBM	0.15	0.8364	+0.0279 [+0.0186, +0.0386]	0	0.772	0.903
BraTS-Africa	0.55	0.7554	+0.0914 [+0.0544, +0.1335]	9	0.898	0.720
BraTS-Africa	0.45	0.7661	+0.0807 [+0.0484, +0.1175]	8	0.878	0.731
BraTS-Africa	0.35	0.7753	+0.0714 [+0.0424, +0.1048]	5	0.837	0.751
BraTS-Africa	0.25	0.7806	+0.0661 [+0.0393, +0.0975]	4	0.778	0.767
BraTS-Africa	0.15	0.7787	+0.0680 [+0.0417, +0.0987]	4	0.673	0.777

SAM 2.1-L, whole tumour. The detector-stage term uses the same oracle-box arm at every threshold; its interval is a paired percentile bootstrap (20 000 draws, seed 1337). Collapses are patients below 0.5 Dice. Precision and recall are slice-level detection figures pooled over the cohort.

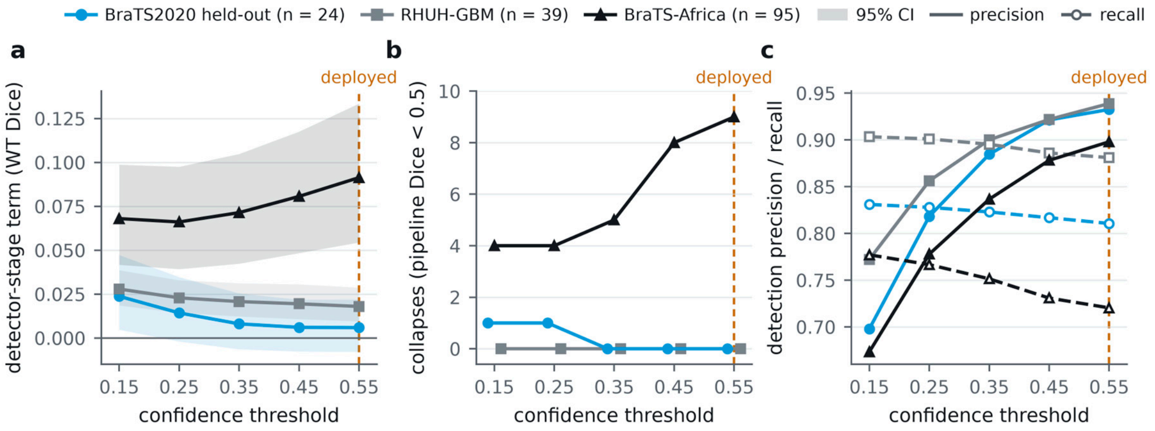

Figure S3. The confidence-threshold sweep on all three cohorts. (a) The detector-stage term with its 95% bootstrap interval; (b) collapses (pipeline Dice below 0.5); (c) precision (solid) and recall (dashed). The deployed threshold of 0.55 is marked.

S17.2 Boundary agreement of the detector-free arm

The detector-free arm was re-run at adapter seed 42 with its masks kept and scored with the same boundary code, slice set, tolerance and empty-mask rule as the two box arms (Section S2). Its whole-tumour, tumour-core and enhancing-tumour Dice matched the stored results to six decimals on every patient. As a check of the convention, the pipeline volume rebuilt from the cached boxes reproduces the stored pipeline HD95 and NSD of Table S2 on all 63 held-out and RHUH-GBM patients. A component that does not touch the reference is counted in three dimensions (26-connectivity). Table S16 gives the results.

Table S16. Boundary agreement of the detector-free arm.

cohort	n	detector-free median HD95, mm [IQR]	mean NSD, oracle-box/pipeline/detector-free	HD95 > 20 mm, oracle-box	pipeline	detector-free	detector-free patients with a detached component	detached share of predicted volume, median, HD95 > 20 mm
BraTS2020 held-out	24	7.03 [2.96–29.57]	0.765/0.745/0.873	1/24	1/24	7/24	23/24	2.4%
RHUH-GBM	39	5.10 [1.86–28.77]	0.765/0.714/0.907	0/39	4/39	13/39	36/39	2.2%
BraTS-Africa	95	12.17 [3.61–29.97]	0.683/0.580/0.798	5/95	20/92	37/95	90/95	4.6%

Beside the two box arms; whole tumour, NSD at 2 mm. Counts of HD95 above 20 mm are over the patients with a predicted surface. The last two columns explain the detector-free arm's tail: detached components occur in almost every patient and in every patient above 20 mm, and they are a small share of the predicted volume. Keeping only the largest three-dimensional component of each detector-free mask (26-connectivity) and rescoring with the same code gives a median HD95 of 3.30, 2.00

and 4.00 mm, HD95 above 20 mm in 1/24, 1/39 and 9/95 patients and a mean NSD of 0.889, 0.938 and 0.834. This filter was applied to no arm in the main analysis. The largest-component filter changes the detector-free arm's Dice little (0.9179, 0.9385 and 0.8899; median removed volume 0.7%, 0.4% and 1.0%). Applied to the pipeline it gives a Dice of 0.8622, 0.8431 and 0.7283, a median HD95 of 5.24, 6.71 and 10.82 mm and HD95 above 20 mm in 0/24, 2/39 and 24/92 patients, so it does not help the pipeline on BraTS-Africa. In multifocal disease the filter would also remove true tumour.

S17.3 RHUH-GBM before and after the preprocessing correction

The fault lay in two rules that took skull-stripped background to be exactly zero (Section S8): the intensity normalisation of the box arms, with its verbatim mirror in the detector-free arm's input, and the slice-admission rule shared by all arms. RHUH-GBM is released z-score normalised with a negative constant background, so on that cohort both rules thresholded at the volume mean. The other two cohorts are unaffected, the corrected rules being bit-identical wherever the background is zero. The fourth arm was run only on the corrected volumes; re-running it reproduced the stored values on all 39 patients. Table S17 lists every arm before and after. Two of the 39 patients (RHUH_0016 and RHUH_0024) had been treated before the preoperative scan. Without them the detector-stage term is +0.0156 [+0.0086, +0.0250] and its contrast against the held-out set +0.0097 [−0.0080, +0.0262] (bootstrap p = 0.27), computed with the scheme of Section 2.6 and fresh resamples.

Table S17. RHUH-GBM before and after the preprocessing correction.

arm or term	before	after
oracle-box, SAM 2.1-L	0.8963	0.8643
pipeline, SAM 2.1-L	0.8271	0.8463
oracle-box, SAM 1 ViT-B	0.8795	0.8178
pipeline, SAM 1 ViT-B	0.8121	0.8028
detector-free, adapter seed 42	0.8508	0.9314
detector-free, adapter seed 52	0.8788	0.9374
detector-free, adapter seed 62	0.8696	0.9353
fourth arm	—	0.9512
detector-stage term	+0.0692	+0.0180
residual	−0.0455	+0.0671
total gap	+0.0237	+0.0850
detector-stage term, contrast vs. held-out [95% CI]	+0.0633 [+0.0191, +0.1221]	+0.0120 [−0.0062, +0.0295]

Every arm on RHUH-GBM (n = 39), whole-tumour Dice; terms at detector seed 1337 and adapter seed 42. "Before" means every arm uncorrected; Table S10 instead compares the detector-free arm before and after against the corrected box arms, and keeps the corrected slice-admission rule in its published-preprocessing column, changing only the normalisation. That is why its residual and gap differ from the "before" column here, and why its detector-free means can differ in the fourth decimal (0.8787 against 0.8788 at adapter seed 52).

Before the correction the residual was negative and the detector-stage contrast excluded zero; both readings were consequences of the mask, not of the cohort.

S17.4 Cohort characteristics

The main-text cohort table gives the main features; Table S18 adds the remaining ones and the pair-wise comparisons with the held-out set. The contrast index is the median intensity inside the whole-tumour reference minus the median in normal-appearing brain (brain minus whole tumour), divided by the interquartile range of normal-appearing brain, for each input channel. The brain is every voxel that differs from the background value in any channel, the background being the mode of the eight corner voxels. The index is unchanged by any linear rescaling of the intensities, which is what makes the z-score normalised RHUH-GBM comparable with the two raw-intensity cohorts. All p values are descriptive and unadjusted.

Table S18. Cohort characteristics measured on the released images.

feature	BraTS2020 held-out	RHUH-GBM	BraTS-Africa	Kruskal–Wallis p	RHUH-GBM vs held-out, p	BraTS-Africa vs held-out, p
WT volume, cm^3	99.8 (69.3, 147.9)	111.0 (58.3, 159.2)	162.9 (77.6, 232.1)	0.001	0.983	0.008
TC volume, cm^3	42.3 (24.1, 70.5)	31.2 (12.8, 51.0)	45.1 (17.3, 112.5)	0.048	0.091	0.585
ET volume, cm^3	14.7 (3.8, 23.5)	18.0 (8.5, 34.4)	31.1 (14.0, 64.6)	9.5×10^{-4}	0.260	0.002
brain volume, cm^3	1440 (1380, 1534)	1487 (1405, 1546)	1543 (1441, 1667)	0.011	0.305	0.009
slices containing WT	68 (59, 77)	66 (54, 78)	78 (60, 88)	0.005	0.915	0.024
WT area per positive slice, mm^2	1530 (1154, 1824)	1452 (923, 2068)	2049 (1305, 2754)	0.004	0.983	0.014
WT connected components	3.5 (2.0, 8.2)	1.0 (1.0, 2.0)	1.0 (1.0, 2.0)	5.4×10^{-7}	1.6×10^{-5}	1.8×10^{-7}
contrast index, FLAIR	2.00 (1.44, 3.03)	2.15 (1.51, 2.63)	1.32 (0.94, 1.68)	8.5×10^{-7}	0.860	5.0×10^{-4}
contrast index, T1ce	−0.27 (−0.66, 0.02)	−0.37 (−0.58, −0.03)	0.21 (−0.02, 0.42)	1.8×10^{-8}	0.772	2.7×10^{-4}
contrast index, T2	1.38 (1.04, 1.92)	1.64 (1.30, 2.62)	1.74 (1.20, 2.36)	0.220	0.099	0.120

Median (interquartile range) over patients, Kruskal–Wallis test across the three cohorts and Mann–Whitney tests against the held-out set.

The verbatim source statements behind the reported rows of the main-text cohort table are included in the released archive with their URLs.

Case-level characteristics. The per-case grade and age of the held-out patients come from the files distributed with BraTS2020 (name_mapping.csv, survival_info.csv), and those of RHUH-GBM from its clinical table on TCIA; Section S15 records where they were obtained. The held-out set has 17

HGG, 7 LGG patients; age is released for 12 of them. Within it, the mean detector-stage term is +0.0056 for high-grade and +0.0068 for low-grade patients, too few for a comparison. Of the 39 RHUH-GBM patients used, all are glioblastoma, WHO grade 4 (35 IDH wild-type, 4 IDH-mutant), and two had surgery and chemoradiotherapy before the scan used here.

S17.5 Effect sizes

Table S19 gives the effect sizes of every term within each cohort.

Table S19. Effect sizes of every term.

cohort	term	mean	median	Hodges–Lehmann [95% CI]	rank-biserial r	Cohen's d_z	patients > 0
BraTS2020 held-out	detector-stage	+0.0059	+0.0050	+0.0035 [−0.0056, +0.0110]	0.21	0.16	13/24
BraTS2020 held-out	residual	+0.0517	+0.0471	+0.0502 [+0.0354, +0.0671]	0.96	1.33	22/24
BraTS2020 held-out	total gap	+0.0576	+0.0493	+0.0504 [+0.0388, +0.0648]	0.98	1.16	22/24
BraTS2020 held-out	prompt	+0.0184	+0.0129	+0.0143 [+0.0098, +0.0219]	1.00	1.01	24/24
BraTS2020 held-out	model	+0.0701	+0.0584	+0.0659 [+0.0501, +0.0847]	1.00	1.76	24/24
RHUH-GBM	detector-stage	+0.0180	+0.0095	+0.0129 [+0.0072, +0.0184]	0.75	0.58	30/39
RHUH-GBM	residual	+0.0671	+0.0691	+0.0667 [+0.0567, +0.0784]	0.93	1.63	38/39
RHUH-GBM	total gap	+0.0850	+0.0830	+0.0854 [+0.0722, +0.0992]	0.96	1.85	38/39
RHUH-GBM	prompt	+0.0198	+0.0088	+0.0139 [+0.0079, +0.0209]	0.99	0.73	38/39
RHUH-GBM	model	+0.0869	+0.0840	+0.0842 [+0.0719, +0.0979]	1.00	2.29	39/39
BraTS-Africa	detector-stage	+0.0914	+0.0164	+0.0315 [+0.0163, +0.0649]	0.68	0.46	74/95
BraTS-Africa	residual	+0.0362	+0.0569	+0.0582 [+0.0447, +0.0705]	0.65	0.23	79/95
BraTS-Africa	total gap	+0.1275	+0.0900	+0.1011 [+0.0882, +0.1206]	1.00	1.10	95/95
BraTS-Africa	prompt	+0.0379	+0.0136	+0.0188 [+0.0140, +0.0270]	0.96	0.48	91/95
BraTS-Africa	model	+0.0741	+0.0718	+0.0773 [+0.0668, +0.0878]	0.86	0.72	88/95

Per cohort. Hodges–Lehmann is the median of the Walsh averages of the per-patient differences, with a percentile bootstrap interval (20 000 draws, seed 1337). Rank-biserial r is the matched-pairs rank-biserial correlation. d_z is the mean over the standard deviation of the per-patient differences, given for reference although the skew of the detector-stage term makes it a poor summary there.

Between cohorts, Cliff's delta for the detector-stage term is +0.36 [+0.15, +0.57] for BraTS-Africa against the held-out set, +0.26 [−0.03, +0.54] for RHUH-GBM against the held-out set and +0.17 [−0.03, +0.36] for BraTS-Africa against RHUH-GBM. The two-sample Hodges–Lehmann shifts are +0.0186 [+0.0067, +0.0353], +0.0095 [−0.0009, +0.0197] and +0.0074 [−0.0013, +0.0178]. Before adjustment, the bootstrap p of the two primary contrasts is 1.0×10^{-4} and 0.181, and the Mann–Whitney p is 0.006 and 0.080. Bias-corrected and accelerated intervals for the two primary contrasts, with the acceleration from each cohort's jackknife, are [+0.0492, +0.1353] and [−0.0077, +0.0281].

On BraTS-Africa the detector-stage term is 72% of the mean gap [47%, 98%] (paired bootstrap of the ratio of means) and 122% of the increase in the gap over the held-out set [77%, 193%] (cohorts resampled independently); a share above 100% means that the residual is smaller there than in domain. Both use 20 000 resamples at seed 1337.

S17.6 Precision of the 24-patient in-domain set

Given the observed spread of the in-domain detector-stage term, the smallest term detectable at 80% power and two-sided $\alpha = 0.05$ is 0.0226 Dice (t-based); for the total gap it is 0.0298. For the primary contrasts the corresponding normal-approximation values, descriptive and computed from the observed spread, are 0.0612 for BraTS-Africa and 0.0257 for RHUH-GBM. These are design-precision figures, not tests: they rescale the interval widths. What the RHUH-GBM result allows is read from its interval, which is compatible with increases of up to +0.0295 (+0.0320 under the Bonferroni interval). Removing each held-out patient in turn keeps the BraTS-Africa contrast between +0.0818 and +0.0913 and the RHUH-GBM contrast between +0.0085 and +0.0179; no single patient changes either reading. These figures describe sampling precision only. The bias from these patients having been used in model selection is a separate matter, treated in Sections S5 and S10.

S17.7 Further detail on the decomposition and the sub-regions

The ordering of the two terms across cohorts. The detector-stage term is larger on both external cohorts than on the held-out set, by 0.012 and 0.085 Dice, and never changes sign; the residual is not ordered that way at all, rising on RHUH-GBM and falling on BraTS-Africa. In the mean the two external cohorts also differ from each other, which their separate contrasts against the held-out set do not by themselves establish; the interval on that exploratory difference excludes zero at both detector seeds (note to Table 4), while the rank-based estimators do not separate them (Section S17.5). The residual is reported under the same analysis rather than in raw form, since intervals on one term and not the other would make the comparison between them an artefact of the reporting.

Sub-region headroom across adapter seeds. The oracle-box arm was scored on tumour core and enhancing tumour as well as on whole tumour, against the detector-free arm on the same regions; Table 7 gives the result. For tumour core the three adapter seeds span +0.1233 to +0.1323 in domain, +0.1312 to +0.1573 on RHUH-GBM and +0.1376 to +0.1565 on BraTS-Africa, so the headroom re-

ported is the largest of the three on both BraTS cohorts. For enhancing tumour the spread is wider and changes sign in domain, +0.0387 to −0.0479 there against −0.0079 to −0.1192 on RHUH-GBM and +0.0901 to +0.0119 on BraTS-Africa, so no ordering of the two arms on that region is claimed and the argument below rests on the tumour-core rows. The mean volume errors, +3.3%, +97.6%, +5.2%, +60.7%, +15.7% and +54.8%, are given beside the medians so that the direction of the enhancing-tumour result can be read under either estimator, the larger means being carried by the few cases in which a nearly empty reference inflates the relative error.

ET is capped by the prompt, under the protocol studied here (Figure 4). What follows concerns a single tight axis-aligned box, the prompt this pipeline emits, and bounds neither dilated nor multi-box prompts. ET is not a thin enhancing rim around a necrotic region but most of the core — a median 61%, 72% and 77% of core volume across the cohorts, the non-enhancing part a median 39%, 28% and 23%. What makes the two boxes coincide is not that share, however, but whether the enhancing tissue reaches the core's extreme voxel in every direction: a rim of enhancement around a necrotic centre has exactly the tight box of the core it encloses, and so does a bulky enhancing mass that no non-enhancing tissue extends beyond. The enhancing mask itself encloses the non-enhancing part on only 3.0%, 51.6% and 32.7% of enhancing slices. Counting slices on which the two tight boxes are **identical**, that holds on 48.1% of the 978 slices carrying both in the held-out set, 99.4% of 1 700 in RHUH-GBM and 85.2% of 4 929 in BraTS-Africa, at mean box IoU 0.806, 0.999 and 0.955. RHUH-GBM's figure is partly a property of its labelling: there the core is necrosis and enhancing tumour only, the non-enhancing tumour having been assigned to the peritumoural label (Section S8), so the enhancing box encloses the core almost by construction and the rate is not comparable with the two BraTS cohorts.

Fragmentation does not explain the cap: enhancing tumour has more than one connected component on 56.7%, 45.7% and 46.1% of the slices carrying it, but so does whole tumour on 55.4%, 40.5% and 35.7% of its own. What singles enhancing tumour out is that its box is also that of a larger structure the segmenter can return in its place — and does. On RHUH-GBM the two boxes are equal on every slice for 31 of 39 patients, and over those 31 the ET-prompted mask scores 0.909 against TC but only 0.730 against ET (Section S15). The substitution shows across all three cohorts: the median ratio of ET-prompted to TC-prompted predicted volume is 0.979, 1.000 and 1.000, and the ET-prompted prediction matches the *tumour-core* reference to within a median of 10.4%, 6.8% and 8.6%. Equality is sufficient but not necessary: the held-out set, where no patient meets the every-slice criterion (0 of 22), behaves the same at a mean box IoU of 0.806.

Slices carrying no non-enhancing core make the boxes coincide by definition rather than as evidence; excluding them the coincidence rates are 44.5%, 99.1% and 80.6% rather than 48.1%, 99.4% and 85.2%, lower but leaving the conclusion unchanged. Where the boxes coincide, a detector constrained

to the same tight-box target cannot resolve the ambiguity by better localisation: this bounds accuracy, not ingenuity.

The detector-free arm is not handicapped by its region-name prompts, two of which are outside the model's training query vocabulary. The in-vocabulary alternative its own source project recommends, composing the regions by union of the three raw BraTS classes, is worse on the held-out set under an otherwise identical path — whole tumour 0.9189 to 0.6996, a paired +0.2193 [+0.1711, +0.2677] in favour of the deployed protocol, and tumour core 0.7590 to 0.7273, +0.0317 [+0.0049, +0.0565], enhancing tumour bit-identical — so its reported figures are the favourable ones.

Trained TC detectors claim part of that headroom and expose the rest. The same architecture trained on tumour-core boxes at three seeds reaches run detection F1 of 0.7688, 0.7657 and 0.7590 against 0.8185 for whole tumour, at the logging threshold of 0.25 on the detector's own test split. The two figures are not like-for-like: the tumour-core split carries 2 695 tumour-core slices in 4 046 images against 3 768 whole-tumour slices in 5 622, and run 37 was selected as the highest test F1 of the 37 ablation runs (Section S10). Retraining that same configuration at a second seed gives 0.8116, so the whole-tumour detector reads 0.8185 and 0.8116 against 0.7688, 0.7657 and 0.7590 on tumour core — a gap of about 0.05 against the 0.0187 this architecture varies by between seeds (Section S13). Box IoU is almost unchanged (0.8246, 0.8182 and 0.8227 against 0.8455), so it is the finding of the sub-region that degrades and not the bounding of it. Fed into the same pipeline these detectors score 0.7984–0.8163, 0.8918–0.8952 and 0.6569–0.7147 on the three cohorts, retaining 30% to 43%, 90% to 92% and −25% to 12% of the headroom of Table 7 (Section S16, Table S13). Closing the decomposition on this region rather than reporting the headroom alone gives a detector-stage term of +0.0750 to +0.0930 in domain, +0.0122 to +0.0156 on RHUH-GBM and +0.1373 to +0.1951 on BraTS-Africa across those seeds. Two whole-tumour readings do not survive it: the in-domain term excludes zero at every seed, and RHUH-GBM falls below the held-out set rather than above it, the contrast excluding zero in that direction. BraTS-Africa is the weaker of the two contrasts on this region, excluding zero at two of the three detector seeds and spanning it at the third, +0.0623 [−0.0079, +0.1274] (Section S16, Table S14).

The prompt is therefore workable for TC while the detector that would exploit it is only partly within reach, and on BraTS-Africa perhaps not at all: against the detector-free model's 0.6956 one seed falls below, one is within 0.001 of it and one is above, the oracle-box arm staying above all three. That comparator is adapter seed 42, the weakest of the adapter's own three on this cohort: against their mean of 0.7067 two of the three pipelines fall below, and against their best of 0.7144 two do, the third exceeding it by 0.0003. No comparator separates the two systems there, and these runs do not settle the sign of the end-to-end comparison out of domain, though the decomposition still localises the problem, the detector-stage term costing the pipeline more than the residual returns to it. Detectors differing by 0.0098 in run detection F1, seed noise under any reading, span 0.0578 Dice there (Figure

S2), the pipeline falling close to zero on a few patients whose identity is not stable across seeds rather than degrading gradually.

Instability is not specific to the staged design. The detector-free model at three LoRA seeds under an identical protocol gives tumour-core spreads of 0.0090, 0.0261 and 0.0188 on the held-out set, RHUH-GBM and BraTS-Africa, against 0.0180, 0.0033 and 0.0578 for the pipeline. With three seeds per design these are ranges rather than variances, and neither design is consistently the more variable. In both, variation is carried by a tail of catastrophic cases rather than by a uniform shift, and neither should be compared with the other on a single training run on out-of-domain data.

S17.8 The BraTS-Africa collapses and a coverage check from the detector's output

The coverage diagnostic of Section 3.4 counts tumour-bearing slices, so it needs the reference and cannot be deployed. A check that uses only the detector's output was therefore defined on the boxes already computed at the deployed threshold of 0.55. Its rule was fixed before it was applied to the cohorts, but after the collapses had been seen. For each patient, over the slices admitted for inference (Section S14), it takes the longest run of consecutive slices carrying a box. A patient is flagged when that run is shorter than 27 slices, the shortest run in the held-out set, so that no held-out patient is flagged. A second rule, a total of fewer than 27 boxed slices, was fixed the same way as a sensitivity check. Intervals on sensitivity are exact (Clopper–Pearson).

On BraTS-Africa the run rule flags 17/95 patients (10.8% to 27.1%) and catches 8 of 9 collapses [52%, 99.7%] with 9 false alarms among the other 86 patients (10.5%, 4.9% to 18.9%); 8/17 flags are collapses (47%, 23% to 72%); the count rule catches 6 of 9 [30%, 93%] with 5 false alarms. Neither rule flags any of the 39 RHUH-GBM patients, none of whom collapses. The threshold rests on 24 patients and the rules were scored on the cohorts that motivated them, so these figures need prospective confirmation. On the 1 mm grid the cut-off is 27 mm. Resampling the held-out patients moves it: it stays at 27 in 64% of resamples, and at the next value, 36, the rule would catch 9 of 9 with 15 false alarms. The false alarms have small tumours, a median whole-tumour volume of 65.4 cm^3 against 164.9 cm^3 in the other patients (Mann–Whitney $p = 0.003$), and the collapses smaller still (37.6 cm^3), so a run length in slices penalises small tumours by construction.

To ask what the collapses have in common, the nine were compared with the other 86 patients on measures taken from the released images (Table S20). Brain volume and the FLAIR contrast index are those of Section S17.4; tumour position is the mean index of the slices containing whole tumour, placed between the lowest and highest brain-containing slices (0 lowest, 1 highest); the field of view counts as truncated when the brain mask reaches the first or last axial slice or the in-plane edge. Medians, collapses against the others: brain volume 1407 against 1559 cm^3 (Mann–Whitney $p = 0.014$); brain-containing slices 134 against 140 ($p = 0.19$); tumour position 0.30 against 0.57 ($p = 0.017$); FLAIR contrast index 0.71 against 1.34 ($p = 0.003$); truncated field of view in 4 of 9 against 17 of 86.

On the tumour-bearing slices of each patient (a slice not admitted for inference scoring 0), the median of the detector's highest score per slice is 0.18 in the collapses against 0.94 in the others ($p = 3.2 \times 10^{-6}$), and in a typical collapse 38% of those slices score below 0.15: the detector is not narrowly missing the threshold. The p values are descriptive, with no correction for the five measures.

Lesion location was classed with an automatic rule: a tumour counts as low and midline, a pattern that suggests an infratentorial location, when most of its voxels lie in the lowest 30% of the brain-containing slices and within 25 mm of the mid-sagittal plane. The rule finds this in 8 of 95 BraTS-Africa patients, in 4 of 9 collapses against 4 of 86 others (Fisher $p = 0.002$), in none of the held-out or RHUH-GBM patients, and in 0 of 258 patients of the detector's training split (95% CI upper bound 1.4%). The training-split count uses the processed slice sets because the 258 volumes are not stored; on the 69 patients whose volumes are stored the approximate and the full rule agree. The rule is crude and was not checked by a radiologist.

Table S20. The nine BraTS-Africa collapses.

patient	brain volume, cm³	tumour position	FLAIR contrast index	truncated field of view	low and midline	median top score, tumour slices	tumour slices scoring < 0.15	flagged, run rule	flagged, count rule
BraTS_SSA_00092	1703	0.30	0.09	yes	yes	0.82	16%	no	no
BraTS_SSA_00119	1406	0.62	0.96	no	no	0.44	9%	yes	no
BraTS_SSA_00129	878	0.63	0.90	yes	no	0.18	38%	yes	yes
BraTS_SSA_00146	1486	0.17	1.51	yes	yes	0.08	77%	yes	yes
BraTS_SSA_00158	1444	0.23	1.18	no	yes	0.36	28%	yes	yes
BraTS_SSA_00180	1407	0.30	0.34	yes	no	0.12	62%	yes	yes
BraTS_SSA_00213	1510	0.60	0.71	no	no	0.76	3%	yes	no
BraTS_SSA_00215	1298	0.24	0.53	no	yes	0.07	60%	yes	yes
BraTS_SSA_00227	1283	0.49	0.66	no	no	0.14	52%	yes	yes

Collapse is pipeline whole-tumour Dice below 0.5 at the deployed threshold. Medians over the other 86 patients are given in the text.

S17.9 Volume error and the slice-knowledge split

Volume error. The relative error of the whole-tumour volume, (prediction − reference)/reference, has a median of −3.4%, +4.0% and −8.1% for the pipeline and −2.3%, +4.1% and −4.6% for the detector-free arm on the three cohorts, with 90th percentiles of +8.3%, +25.5% and +20.7% against +7.1%, +16.9% and +5.1%. Among patients who do not collapse, the error exceeds 20% in absolute value in 2/24, 9/39 and 21/86 patients for the pipeline and 1/24, 3/39 and 2/86 for the detector-free arm.

Slice knowledge. The oracle-box arm and the fourth arm are prompted only on slices that contain the region, so both know which slices contain tumour. Restricting the pipeline and the detector-free arm to those slices splits each term into a slice-knowledge part and a within-slice part: detector-stage term =

(oracle-box − restricted pipeline) + (restricted pipeline − pipeline), and prompt term = (fourth arm − restricted detector-free) + (restricted detector-free − detector-free). The slice-knowledge part of the detector-stage term is +0.0006 [+0.0001, +0.0016], +0.0020 [+0.0003, +0.0045] and +0.0034 [+0.0013, +0.0063], and its within-slice part +0.0053 [−0.0083, +0.0212], +0.0160 [+0.0076, +0.0267] and +0.0880 [+0.0518, +0.1299]. For the prompt term the two parts are +0.0068 [+0.0034, +0.0115], +0.0080 [+0.0040, +0.0136] and +0.0119 [+0.0076, +0.0171], and +0.0116 [+0.0073, +0.0165], +0.0119 [+0.0068, +0.0184] and +0.0259 [+0.0156, +0.0403]. Intervals are paired percentile bootstraps (20 000 resamples, seed 1337). The pipeline masks were rebuilt from the stored detector outputs and reproduce the stored Dice to six decimals.
